# Gene expression profiling in whole blood identifies distinct biological pathways associated with obesity

**DOI:** 10.1186/1755-8794-3-56

**Published:** 2010-12-01

**Authors:** Sujoy Ghosh, Robert Dent, Mary-Ellen Harper, Shelby A Gorman, Joan S Stuart, Ruth McPherson

**Affiliations:** 1 Biomedical Biotechnology Research Institute, North Carolina Central University, Durham, USA; 2 Ottawa Hospital Weight Management Clinic, Ottawa Hospital, Ottawa, Canada; 3 Department of Biochemistry, Microbiology and Immunology, Faculty of Medicine, University of Ottawa, Ottawa, Canada; 4 GlaxoSmithKline, Research Triangle Park, USA; 5 University of Ottawa Heart Institute, Ottawa, Canada

## Abstract

**Background:**

Obesity is reaching epidemic proportions and represents a significant risk factor for cardiovascular disease, diabetes, and cancer.

**Methods:**

To explore the relationship between increased body mass and gene expression in blood, we conducted whole-genome expression profiling of whole blood from seventeen obese and seventeen well matched lean subjects. Gene expression data was analyzed at the individual gene and pathway level and a preliminary assessment of the predictive value of blood gene expression profiles in obesity was carried out.

**Results:**

Principal components analysis of whole-blood gene expression data from obese and lean subjects led to efficient separation of the two cohorts. Pathway analysis by gene-set enrichment demonstrated increased transcript levels for genes belonging to the "ribosome", "apoptosis" and "oxidative phosphorylation" pathways in the obese cohort, consistent with an altered metabolic state including increased protein synthesis, enhanced cell death from proinflammatory or lipotoxic stimuli, and increased energy demands. A subset of pathway-specific genes acted as efficient predictors of obese or lean class membership when used in Naive Bayes or logistic regression based classifiers.

**Conclusion:**

This study provides a comprehensive characterization of the whole blood transcriptome in obesity and demonstrates that the investigation of gene expression profiles from whole blood can inform and illustrate the biological processes related to regulation of body mass. Additionally, the ability of pathway-related gene expression to predict class membership suggests the feasibility of a similar approach for identifying clinically useful blood-based predictors of weight loss success following dietary or surgical interventions.

## Background

While excess energy intake and declining energy expenditure are clearly important contributors, individual susceptibility to obesity is also strongly influenced by genetic factors. Twin, adoption, and family studies have indicated that 40-70% of inter-individual variation in body mass index (BMI) is heritable [1,2]. A compendium of evidence for the genetic bases of obesity have been accrued from single-gene mutation studies, Mendelian inheritance patterns, transgenic and knockout murine models, animal and human quantitative trait loci (QTL), candidate-gene association studies, and genome scan linkages and have been incorporated into the Obesity Gene Map database [3]. Also recently, a number of genome-wide association studies (GWAS) have demonstrated associations of single-nucleotide polymorphisms (SNPs) to qualitative and quantitative indices of adiposity in several populations [2,4-10]. A combination of independent studies and meta-analysis of existing GWAS data have implicated a total of 18 genetic loci as relevant for body weight regulation to date [11].

In addition to DNA sequence variants, genetic influences are also manifested through differences in gene transcription, leading to differential messenger RNA levels. While such differences might be expected to occur in biologically relevant tissues (muscle and adipose tissue in obesity, for example), several recent studies have demonstrated an alteration in the peripheral blood transcriptome in diseases of non-hematologic origin. These include disorders such as chronic fatigue syndrome, schizophrenia and colon cancer [12-17]. Additionally, the blood transcriptome has also been found to be responsive to diverse environmental and socio-economic stimuli including ionizing radiation in cancer therapy, benzene exposure, socio-economic status, etc. [18-21]. These findings raise the intriguing possibility that blood transcriptome profiles might provide a valid biological readout for otherwise hard to study disease processes in humans and additionally generate information of high predictive and diagnostic content. In line with this argument, we postulated that differences in transcript abundance might also occur in blood from obese subjects compared to lean subjects, as a consequence of either pre-existing genetic variations, or as an adaptive response to obesity, independent of the genetic background. To test this hypothesis, we have carried out transcriptional profiling of peripheral blood from obese subjects and well-matched lean controls and conducted enrichment analysis to identify biological pathways that are preferentially associated with obesity. Our study demonstrates significant gene expression differences in blood from obese subjects compared to lean controls, particularly along the lines of differential expression of genes in key metabolic pathways regulating cell survival, protein synthesis and energy harvest. These findings are important on three levels. First, our results demonstrate the importance of blood as a biologically informative tissue in the elucidation of the obese state. Second, as differences in gene expression are often driven by sequence variants in gene regulatory regions, our study provides a mechanism for the selection of obesity-associated candidate genes for the determination of possible regulatory sequence variants. Finally, the identification of adiposity related gene expression differences in a clinically accessible tissue such as blood leads the way for the determination of biomarkers of weight regulation that could be implemented in a clinical setting.

## Results

### Phenotypic characterization of study subjects

Demographic and phenotypic characteristics of the subjects included in the current study are shown in Table 1. The obese and lean subjects showed statistically significant differences (p < 0.05 level) in almost all metabolic parameters tested with the exception of cholesterol, LDL-cholesterol and thyroid stimulating hormone status. Also, levels of glycated haemoglobin (HbA1c), insulin and fasting glucose were statistically significantly different but within normal clinical ranges in both groups. There were 53% (9/17) and 70% (12/17) females in the obese and lean groups, respectively. Both cohorts were closely matched for age and hormonal status (6/12 lean and 5/9 obese women were postmenopausal).

**Table 1:** Demographic and phenotypic characteristics of the study population.

*Variable*	*Obese*	*Lean*	*p-value*
**N**	17	17	-
**Female (%)**	9(53%)	12(70%)	-
**Age (yrs)**	52.2(10.2)	47(9.3)	0.1270
**BMI at baseline (kg/m2)**	44.8(8.1)	20.7(1.9)	0.0000
**BP, diastolic (mm Hg)**	84.4(8.2)	69.8(8.7)	0.0001
**BP, systolic (mm Hg)**	143.0(11.8)	119.1(12.3)	0.0000
**Waist circumference (cm)**	125.7(15.1)	77.7(7.3)	0.0000
**Weight (kg)**	127.6(26.5)	58.8(8.3)	0.0000
**Body fat (%)**	45.9(7.3)	22.0(6.9)	0.0000
**Fat free mass (kg)**	67.4(10.3)	45.7(7.9)	0.0000
**Fat mass (kg)**	59.5(19.9)	12.9(4.2)	0.0000
**Fasting glucose (mmol/L)**	6.3(2.2)	4.8(0.4)	0.0102
**Insulin (pmol/L)**	99.8(56.7)	27.6(11.0)	0.0001
**HbA1c (%)**	5.9(0.8)	5(0.4)	0.0002
**HDL-cholesterol (mmol/L)**	1.0(0.2)	1.6(0.3)	0.0000
**LDL-cholesterol (mmol/L)**	3.2(0.6)	3.3(0.9)	0.6441
**Cholesterol (mmol/L)**	5.1(0.8)	5.4(0.9)	0.2673
**Triglycerides (mmol/L)**	1.9(1.8)	0.9(0.5)	0.0399
**Thyroid stimulating hormone (mU/L)**	2.6(1.3)	1.9(1.6)	0.2073

Values for continuous measures are shown as average (standard error); p-values are obtained from two-sided Student's t-test, assuming unequal variances between groups.

### Ascertainment of data quality

**Figure 1:**
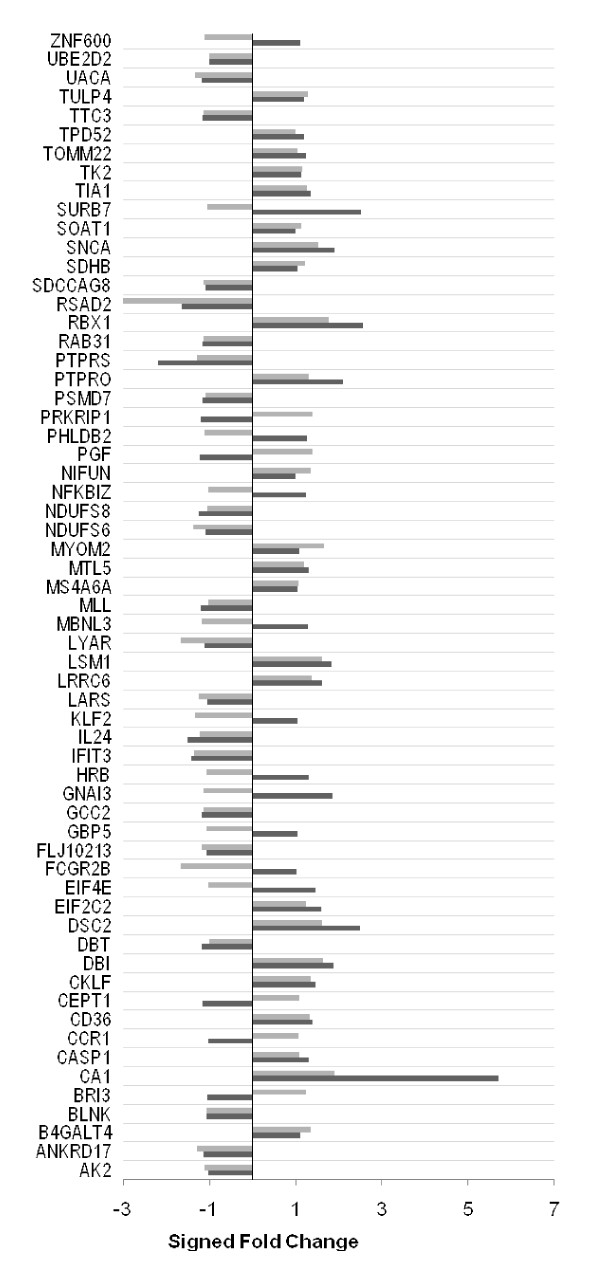
**Comparison of gene expression signals generated by Affymetrix microarrays and quantitative real-time PCR**. Gene expression signals were generated by real-time, quantitative PCR (Taqman, black bars) and oligonucleotide microarrays (Affymetrix, white bars). Overexpression or underexpression of a gene in the obese and lean cohorts is expressed as a log ratio, to the base 2. Affymetrix and Taqman based results for each gene are shown as a stacked bar. For each gene, agreement between the results from the two platforms is indicated when both white and black bars lie on the same side of the zero (0) value on the log ratio axis; conversely, disagreement is indicated when the gray and black bars lie on opposite sides. The overall agreement between the two platforms was 85% (45/53 genes showed agreement in the direction of differential expression).

We ascertained the overall quality of the whole genome expression profiling signals by comparing the Affymetrix microarray generated expression patterns of a subset of 61 genes (with a 20% or greater change in expression between obese and lean cohorts) to expression signals generated by real-time, quantitative PCR (Taqman). The genes selected cover a range of approximately 7 logs (base 2) representing over 100-fold differences in the magnitude of gene expression on Affymetrix microarrays (average log2 signal of 4.66 for protein tyrosine phosphatase, receptor type, S to an average log2 signal of 11.35 for RAB31 gene in the obese cohort). The results are shown in Figure 1. In ~ 75% of genes tested (45/61 genes), the direction of gene expression changes between obese and lean subjects were in agreement between the Affymetrix and Taqman platforms suggesting high reproducibility of gene expression data between the two approaches. Additionally, analysis of muscle and adipose-specific marker gene expression demonstrated no evidence of contamination in the study samples (Additional File 1).

### Principal components analysis of gene expression data

**Figure 2:**
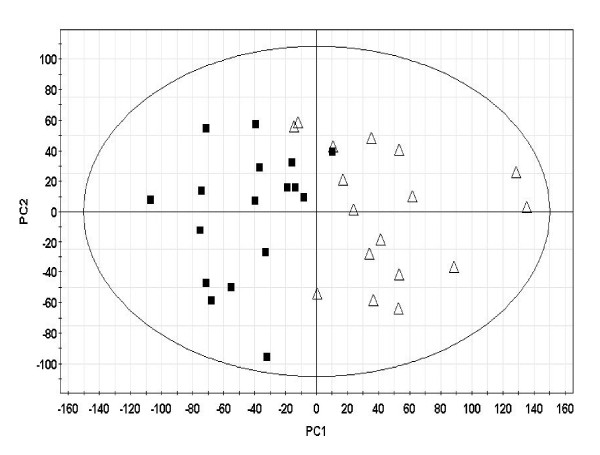
**Multivariate analysis of obese and lean subjects based on gene expression signals**. Principal component analysis (PCA) was performed on lean and obese subjects based on 12128 Affymetrix probe-set signals. A scatterplot of the first two principal components demonstrate a general separation of the obese and lean phenotypes along the first principal component (PC1). Model parameters are as follows: Further details on the PCA model parameters are included in Supplemental Table 2.

We performed multivariate, principal components analysis to determine whether blood gene expression signals were capable of distinguishing between the obese and lean subjects. Figure 2 shows a scatterplot representing the first two principal components based on gene expression profiles from 17 obese and 17 lean subjects. Analysis of the principal component model performance indicated that 27% of the total variance in gene expression was modelled in the first principal component (R2X) with a cross-validated prediction of 22.4%. The cross-validation results indicate that the variability captured in the first component is statistically greater than the significance limit of 2.9% (Additional file 2).

### Identification of differentially expressed genes

Genes showing differential expression between the obese and lean subjects were identified via the Comparative Marker Selection module in GenePattern [22], using the signal-to-noise algorithm for ranking genes. A permutation testing was performed to compute the significance (nominal p-value) of the rank assigned to each gene. A false discovery rate (FDR) was also calculated to control for multiple testing. A total of 12127 probesets were detected above background (set to 50 units) among which 374 probesetes were overexpressed (2-fold or greater) and 75 probesets were underexpressed (2-fold or greater) in the obese samples compared to the leans. The results of the differential gene analysis are presented in Additional Files 3 and 4. Inspection of the gene list showed that a majority of the genes upregulated in the obese subjects were genes known to be selectively expressed in erythrocytes/reticulocytes. These included genes such as carbonic anhydrase, ferrochelatase, synuclein, glycophorin B, etc. This finding is consistent with previous observations of higher red blood cell counts (hematocrit) in obesity [23-26] and provides evidence for the expansion of transcriptionally active reticulocytes in obesity. Conversely, several genes related to immune function showed reduced expression in the obese subjects.

### Pathway analysis of gene expression difference between lean and obese subjects

The transcriptome data was next subjected to bioinformatic pathway analysis by the Gene Set Enrichment Analysis (GSEA) algorithm [27]. The values for the GSEA algorithmic parameters used in the current study are indicated in Additional File 5 and details about the GSEA algorithm have been explained in Materials and Methods. Pathway analysis was conducted either with the Kyoto Encyclopedia for Genes and Genomes (KEGG) metabolic pathway database [28], or a user-created custom database consisting of pathways drawn from several sources (Additional File 6). Pathways were evaluated by their normalized enrichment score (NES), nominal p-values (permuted) and false discovery rates, as described in [29].

#### KEGG Pathway analysis

**Figure 3:**
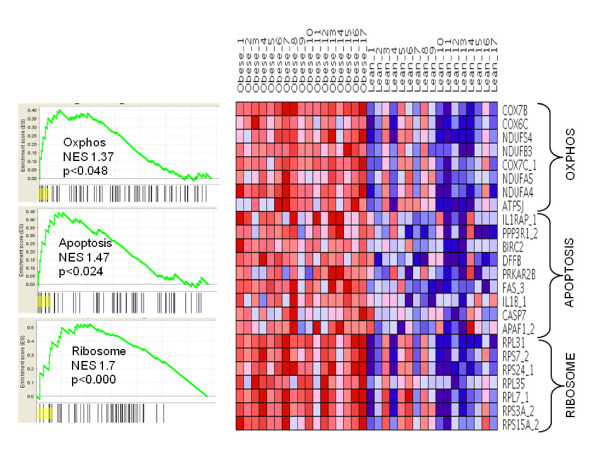
**Gene-set enrichment analysis**. Gene-set enrichment analysis against the KEGG database for differentially enriched pathways in whole blood between obese and lean subjects. Enrichment plots for the 3 pathways upregulated in the obese cohort are shown on the left side with the relative gene positions indicated by the straight lines (line plot) under each graph. Lines clustered to the left represent higher ranked genes in the ranked list. Expression profiles for a subset of genes (shaded in yellow in the line plots) contributing to core enrichment for each pathway are shown to the right as a heatmap. The heatmap compares subject-level gene expression in both obese and lean subjects. Gene expression is normalized for each row. Lower levels of expression are represented in shades of blue and higher expression in red.

Enrichment analysis of gene expression profiles against KEGG pathways identified 5 pathways at p*_permuted _*< 0.05 level (Additional File 7). Notable among them were the *'apoptosis'*, '*ribosome*', and '*oxidative phosphorylation*' pathways. The pathway enrichment plots and expression profiles of a subset of genes contributing significantly to the enrichment of these 3 pathways are collectively shown in Figure 3. A number of genes, including apoptotic protease activating factor 1, baculoviral IAP repeat containing 2, caspase 7, Fas, interleukin 1 beta, interleukin 1 receptor associated kinase 4, etc. contributed to the core enrichment of the 'apoptosis' pathway in the obese subjects (Additional File 8). Enrichment of the ribosome pathway was effected by coordinate upregulation of several ribosomal protein genes (ribosomal protein L31, S7, S24, L35, L7 for example). Several genes involved in the mitochondrial process of electron transfer and ATP synthesis demonstrated increased expression in the obese cohort leading to a significant enrichment of the 'oxidative phosphorylation' pathway in this group. Some of the genes contributing to core enrichment of this pathway included cytochrome c oxidase subunits 6C, 7B and 7C, NADH-coenzyme Q reductase, NADH deyhdrogenase beta subcomplex 3, etc.

#### Custom Pathway analysis

In addition to investigating pathway enrichment based on the KEGG database, we also subjected a set of 'custom' pathways to analysis by GSEA (Additional File 6). GSEA analysis of the custom pathways identified 2 pathways as significantly upregulated in the obese, at a nominal p-value < 5% and FDR < 5%. These were the 'electron transport chain pathway' and the 'erythrocyte/reticulocytespecific_affytechnote' pathways (Additional File 9). The 'electron transport chain pathway' (National Cancer Institute Pathway Interaction Database [30]) is a subset of the KEGG 'oxidative phosphorylation' pathway. The 'erythrocyte/reticulocytespecific_affytechnote' pathway consists of genes reported to be selectively enriched for expression in erythrocytes/reticulocytes (Affymetrix, [31,32]). Identification of this gene-set as an obesity-upregulated pathway further supports our earlier observation of increased expression of individual erythrocyte/reticulocyte specific genes in the obese subjects. Details are provided in Additional Files 10 and 11.

#### Effects of gender on pathway enrichment

Since our study cohort contained both male and female subjects, the contribution of gender to pathway enrichment was investigated. To determine whether pathway ranks were influenced by gender, we carried out independent gene-set enrichment analyses on subgroups comprised of female or male subjects only. We compared the relative ranks of the KEGG pathways in the three analyses as an indication of their sensitivity to gender. 'Apoptosis' was ranked 7^th^, 8^th ^and 3^rd ^and 'oxidative phosphorylation' was ranked 10^th^, 12^th ^and 18^th ^for *All subjects, Females and Males *respectively. The 'ribosome' pathway was the top ranked pathway for *All subjects *and *Females *analysis, but was ranked 27^th ^in the analysis involving the *Males*. We repeated the same subgroup analyses on the custom pathway set and in all cases the 'electron transport chain pathway' and the 'erythrocyte/reticulocytespecific_affytechnote' pathways remained the top 2 ranked pathways for all groups tested. Details are provided in Additional File 12.

#### Effect of cell populations on pathway enrichment

Since whole-blood consists of a mixture of various cell types, we investigated the relation between the observed enrichment in "ribosome", "apoptosis" and "oxidative phosphorylation" pathways in the obese and enrichment of reticulocytes/erythrocytes in obese subjects as previously reported [23-26]. We scaled the gene expression data independently by the expression of 2 erythrocyte-specific transcripts, hemoglobin D (HBD) and erythrocyte membrane protein, band 2 (EMPB2) and subjected the scaled data to gene-set enrichment analysis. Of the original 3 pathways found to be enriched in the obese subjects, the "ribosome" pathway was still the top differentially expressed pathway with both unscaled and scaled data. However, the "apoptosis" and "oxidative phosphorylation" pathways were no longer significantly enriched, with either of the scaled datasets. Pathway enrichment results with scaled data are provided in Additional File 13.

### Class prediction via blood gene expression

We next examined whether biological pathways implicated from gene-set enrichment analysis of the current study could provide a set of mechanism-based gene predictors that would be capable of predicting obese and lean subjects with high accuracy. We created an initial, inclusive set containing all genes (features) belonging to the ribosome, apoptosis or oxidative phosphorylation pathways (183 genes). Since this list was also likely to contain redundant and non-informative genes, we applied two independent feature selection algorithms to identify a smaller set of genes that would be capable of distinguishing between the obese and lean phenotypes with high success rates, based on the metrics specific to the two algorithms used (described in detail in Materials and Methods). A search for overlapping genes scoring high in both algorithms (ranked within the top 20 genes in both) resulted in a set of 11 genes. The logged gene expression signals from the full (183) and filtered (11) gene-sets were then used as inputs into four different classifiers representing distinct algorithmic approaches to classification and prediction. These included the Naive Bayes, Logistic Regression, Random Forests and ZeroR classifiers. A full description of the classifiers is presented in Materials and Methods and Additional File 14. Each classifier was first trained on a randomly selected 66% of the samples and then used to predict the class for the remaining 33% samples. The process was repeated 100 times for each classifier. Classifier performance was evaluated by four parameters (true positive, true negative, false positive and false negative rates). A description of the performance evaluators can be found in Additional File 14. The classifier ZeroR simply predicts the same class for all instances and was used as a baseline classifier. Any classifier should perform significantly better than ZeroR in order to be considered useful. Table 2 compares the performance of the four classifiers with either the full gene-set (183 genes) or the filtered set (11 genes). For each of the four performance evaluators, we plotted the average and standard deviation values for the four parameters over the 100 iterations. Overall, the Naïve Bayes and logistic regression classifiers performed better than the decision-tree based classifier (Random Forests) and all three classifiers performed significantly better than ZeroR. A comparison of the classifier results with the full (183) or filtered (11) gene-set inputs showed that both inputs had similar true positive and false negative rates. Both Naïve Bayes and logistic regression classifiers displayed high sensitivity as indicated by true positive rates close to 1.0. These two classifiers also demonstrated lower false-positive rates with the filtered gene set compared to the full gene set. Additionally, the filtered gene set classifiers displayed higher specificities (true negative rates) compared to the full gene set based classifiers. Based on these results, we found the 11-gene based Naïve Bayes or logistic regression based classifiers to perform better compared to the 183-gene classifiers for predicting class membership. The identities of the 11 genes are shown in Table 3 and appear to be primarily composed of genes from the oxidative phosphorylation and apoptosis pathways.

**Table 2:** Classification of lean and obese subjects

	*True Positive Rate (Sensitivity)*		*False Positive Rate*		*True Negative Rate (Specificity)*		*False Negative Rate*	
**Classifier**	**Full**	**Filtered**	**Full**	**Filtered**	**Full**	**Filtered**	**Full**	**Filtered**
**Naïve Bayes**	0.96 (0.09)	0.93 (0.08)	0.18 (0.16)	0.003 (0.02)	0.82 (0.17)	0.99 (0.02)	0.03 (0.09)	0.06 (0.08)
**Logistic Regression**	0.98 (0.04)	0.95 (0.07)	0.11 (0.16)	0.01 (0.03)	0.89 (0.16)	0.99 (0.03)	0.01 (0.04)	0.05 (0.08)
**Random Forests**	0.95 (0.09)	0.94 (0.11)	0.17 (0.17)	0.10 (0.14)	0.83 (0.17)	0.89 (0.14)	0.05 (0.09)	0.06 (0.11)
**ZeroR**	0.81 (0.39)	0.81 (0.39)	0.81 (0.39)	0.81 (0.39)	0.19 (0.39)	0.19 (0.39)	0.19 (0.39)	0.19 (0.39)

Comparison of classifier performance for predicting obese and lean phenotype with a **full **(183 genes) or **filtered **(11 gene) gene inputs

**Table 3:** Identity of genes constituting the 11-gene classifier.

*ProbesetID*	*GeneName*
202110_at	cytochrome c oxidase subunit VIIb
208746_x_at	ATP synthase, H+ transporting, mitochondrial F0 complex, subunit G
202875_s_at	ATPase, H+ transporting, lysosomal 42kDa, V1 subunit C1
215719_x_at	Fas (TNF receptor superfamily, member 6)
201134_x_at	cytochrome c oxidase subunit VIIc
201783_s_at	v-rel reticuloendotheliosis viral oncogene homolog A
202076_at	baculoviral IAP repeat-containing 2
208737_at	ATPase, H+ transporting, lysosomal 13kDa, V1 subunit G isoform 1
202429_s_at	protein phosphatase 3 (formerly 2B), catalytic subunit, alpha isoform
206752_s_at	DNA fragmentation factor, 40kDa, beta polypeptide
213052_at	protein kinase, cAMP-dependent, regulatory, type II, alpha

## Discussion

Our study demonstrates significant gene expression differences in whole blood from age-matched obese and lean subjects of Northern European White genetic ancestry. These differences further lead to the identification of differentially enriched biological pathways in obesity and lead to an increased appreciation and understanding of genomic changes in whole blood related to body mass expansion. The current study is not designed to resolve whether the observed transcriptional differences are causal or caused, i.e. whether the differences in gene expression are related to the development of obesity or reflect an adaptive mechanism in response to increased body mass. Although blood is usually not considered to be a target organ for obesity, certain observations are pertinent. First, the physiological role of blood as a sentinel tissue and a systemic integrator of tissue and organ-level perturbations could lead to adaptive responses in response to major metabolic perturbations such as excessive build-up of body mass and the attendant increases in the demand for nutrient and oxygen transport. Secondly, the chronic low-grade tissue inflammation observed in obesity [33] is expected to have a direct effect on circulating leukocytes, including immune dysfunction and apoptosis. Finally, macrophages in blood share many functional and antigenic properties with preadipocytes and adipocytes and transcriptome profiles of preadipocytes are reportedly closer to the macrophages than to adipocytes [34]. In this context, our study provides the first detailed investigation of the blood transcriptome in relation to obesity and provides evidence in favor of its dynamic involvement in the process. It is important to note here that the between-group differences in gene expression were usually small and there was considerable heterogeneity in individual gene expression values among subjects in the obese or lean categories. However, the between-group variation exceeded the within-group variation for several genes leading to statistically significant differences between the groups. Additionally, as demonstrated by principal components analysis, blood gene expression profiles were able to distinguish lean subjects from obese subjects even when the subject classes were not exposed a priori (unsupervised clustering). Since gene expression measures were used as input for the PCA analysis, these results suggest that the differences in blood transcript levels between obese and lean subjects were significant and informative enough to cause a separation between the two classes.

The application of pathway analysis provided additional information and insight into the biological processes that are differentially regulated in obese and lean blood samples. Some of the pathways with increased component transcript abundances included the "ribosome", "apoptosis" and "oxidative phosphorylation" pathways. Upregulation of the ribosomal pathway in the obese subjects was due to an increased expression of several ribosomal protein-encoding genes, indicative of enhanced protein synthesis in blood cells, possibly as a consequence of enhanced metabolic demands in the obese state. This observation is consistent with a recent report that links ribosomal RNA synthesis to cellular energy supply through activation of the AMP-activated protein kinase [35]. The presence of increased apoptosis in the obese phenotype has also been well documented in animal and human cell culture models. For example, increased cardiomyocyte apoptosis has been reported in leptin-deficient ob/ob mice and leptin-resistant db/db mice [36]. Prolonged exposure to free fatty acids also have pro-apoptotic effects on human pancreatic islets [37] and circulating cytokines, such as tumor necrosis factor alpha (TNF-α) have been reported to induce apoptosis in cultured human preadipocytes and adipocytes [38]. Our findings now provide evidence for activation of a similar apoptotic program in blood from obese subjects. While the current study does not allow us to pinpoint the cause of the enhanced apoptosis, we speculate that obesity-associated chronic inflammation [39,40] or lipotoxicity are contributing factors. Finally, the observed upregulation of the 'oxidative phosphorylation' pathway in obese subjects is consistent with a response to increased energy demands in obese subjects. Functional and gene expression studies have previously indicated impairment in oxidative phosphorylation and mitochondrial function in subjects with type 2 diabetes compared to controls [29,41,42]. Our findings are consistent with Takamura et al., who demonstrated an upregulation of oxidative phosphorylation genes in the livers of obese, type 2 diabetic patients compared to non-obese diabetics [43]. More interestingly, our findings now point to a similar involvement of energy-harvesting mechanisms in obese blood and provide further evidence in favor of a role for mitochondrial dysfunction in obesity [44,45]. A gender-based sub-analysis demonstrated relative stability of the "apoptosis" and "oxidative phosphorylation" pathway ranks in both genders; in contrast, the "ribosome" pathway differed significantly in rank between females and males, suggesting a gender-specific effect (Additional File 7). Since a majority of genes upregulated in the obese subjects are highly expressed in erythrocytes and reticulocytes, we scaled the gene expression data independently by the expression of two erythrocyte-specific transcripts, hemoglobin D (HBD) and erythrocyte membrane protein, band 2 (EMPB2) and subjected the scaled data to gene-set enrichment analysis. Of the three pathways found to be differentially upregulated in the obese subjects, the "ribosome" pathway remained the top differentially expressed pathway (with the scaled data) whereas the "apoptosis" and "oxidative phosphorylation" pathways were no longer significantly enriched, with either of the scaled datasets. These findings suggest that an increase in erythrocyte/reticulocyte numbers in the obese (differential hematocrit) is a possible explanatory mechanism for the observed increase in transcript levels for "apoptosis" and "oxidative phosphorylation" in the obese subjects. The results for the "ribosome" pathway, in contrast, suggest a significant upregulation of the transcripts for the component genes of this pathway in the obese subjects, even after adjustment for erythrocyte-specific gene expression. We note one caveat to the scaling approach used here for investigating cell number effects. Since the same amount of cRNA was used from each sample for hybridization, the relative enrichment of cell types is expected to have a real effect on gene expression only for genes that are differentially expressed among the cell types (e.g. hemoglobin transcripts that are expressed only in reticulocytes and not lymphocytes). For genes expressed at comparable levels across cell types, the differential cell type representation should not have an effect on expression unless there is a true upregulation or downregulation of these genes between the two groups (although the cellular origin for the differential expression may not be known). Scaling the gene expression data by the expression of reticulocyte/erythrocyte specific genes cannot distinguish between the above two mechanisms of enhanced gene expression and can lead to potentially incorrect conclusions. However, our results clearly demonstrate that inter-individual variations in hematocrit, especially between obese and lean subjects, may affect interpretation of expression data and should be considered as an important co-variate in future studies.

Several recent publications have reported on the successful application of gene expression signatures as classifiers or predictors of phenotypic class, disease progression and therapeutic prognosis, primarily in the area of diagnosis and treatment of several types of cancers [16,46-48]. However, the biological mechanisms linking the predictive genes to the outcomes being predicted are not always clear. This lack of mechanism has often been criticized as a barrier to the clinical utility of the gene predictors. One solution to the problem is to choose gene predictors from biological pathways associated *a priori *with the phenotype or outcome of interest. This approach was pursued in this study and led to the identification of an 11-gene based classifier that could distinguish and predict obese and lean subjects with high accuracy. Our motivation for this exercise was to provide proof-of-concept data to test if blood gene expression patterns can have predictive value in the context of obesity. While such prediction is not necessarily required for distinguishing obesity from leanness, blood based gene biomarkers can significantly advance the clinical management of obesity by, for example, allowing the prediction of weight loss success from diet or bariatric surgery.

One potential downstream application of differential gene expression analysis in whole-blood is the selection of candidate genes with possible regulatory polymorphisms (single nucleotide polymorphisms in promoter regions, for example) that associate with obesity and help explain the observed differences in expression. Comprehensive sequencing of the regulatory regions of such candidate genes are expected to yield additional insights into the genetics of obesity such as the identification of expression QTLs (eQTLs). While a direct subject-level association of gene regulatory polymorphisms to gene expression levels is outside the scope of the current work, we conducted a preliminary analysis of the existence of putative regulatory variants in the 11 gene predictors identified in our analysis. Based on data from the NCBI dbSNP database (Build 131), several genes contained common sequence variants near the 5'-end of the gene spanning a region 2000 bases upstream of the start codon (SNPs rs2515192 and rs3019164 for ATP6V1C1, rs1317775 and rs1318199 for BIRC2, rs11709092 for PRKAR2A, etc.). It is reasonable to speculate that a subset of these upstream sequence variants could influence transcription.

Our study relied on whole-blood collected in PAXgene tubes instead of peripheral blood mononuclear cells (PBMCs), consistent with our ultimate goal of identifying clinically relevant and useful predictors of weight loss success. This procedure, however, has the disadvantage of investigating a relatively heterogeneous cell population where noise could mask gene expression differences in specific cell types. PBMC's, consisting of lymphocytes and monocytes provide a consistent and homogeneous sample for transcriptome analysis. However, the extra fractionation procedure for PBMCs requires a prolonged period before RNA stabilization leading to significant *ex vivo *changes in gene expression profiling [49]. Additionally, compared to whole blood, several cell types including neutrophils, basophils, eosinophils, platelets, reticulocytes and erythrocytes are depleted in PBMCs which lead to loss of important transcription information. On the other hand, PAX samples show a decrease in the number of expressed genes and lower gene expression values with higher variability compared to the PBMCs [50], primarily due to the high abundance of globin transcripts that constitute over 70% of whole blood mRNA [51]. However, the PAXgene system employs an easy way to collect, store, transport and stabilize RNA from whole blood and based on our overall goals, was the method of choice for our analysis. In this context, the ability of gene expression signatures from biologically relevant pathways to accurately classify and predict obese and lean classes, as observed in this study, provides further validation of our approach and suggests future suitability of the PAXgene based whole blood transcriptome for yielding clinically usable biomarkers related to weight regulation. Additional sensitivity could be obtained in future studies via selective reduction of the globin transcript from whole blood RNA samples [52,53].

There are the following limitations to the current study. First, since the study employed whole blood, the relative contribution of the number and transcriptional programs in specific cell types towards the observed gene expression differences cannot be clearly delineated. Second, the relatively small sample sizes reduced the power for detection of subtle differences in expression. Also, due to small sample numbers, we had to rely on cross-validation methods for calculation of prediction errors instead of testing candidate predictors on new samples. The possibility of over-fitting cannot, therefore, be entirely ruled out.

## Conclusions

Gene expression profiling in whole blood demonstrated significant differences in transcript levels that were capable of separating obese and lean phenotypes in multivariate analysis. Gene-set enrichment analysis further identified differences in biological pathways relating to cell survival, protein synthesis and energy harvest between the obese and lean groups. A subset of genes responsible for pathway enrichment also acted as efficient predictors of phenotype (obese or lean) when their expression signatures were used as inputs to Naive Bayes or logistic regression based classifiers. Together, our study is the first to investigate the information content in whole blood in relation to obesity. Our findings demonstrate that the investigation of gene expression profiles from whole blood can inform and illustrate the biological processes related to regulation of body mass. Additionally, the ability of pathway-related gene expression to predict class membership suggests the feasibility of a similar approach to identify blood-based robust predictors of weight loss success in response to dietary and surgical interventions.

## Methods

### Study Subjects

Twenty consecutive obese subjects enrolled in the Ottawa Hospital Weight Management Program at the Ottawa Hospital, Ottawa, with a body mass index (BMI) of 30-50 kg/m2, were recruited for study. All subjects were of Northern European White genetic ancestry. Patients were excluded on the basis of medical conditions possibly affecting whole blood gene expression, including out of normal range thyroid indices (TSH, free T3) at week 1 or week 13, diabetes mellitus treated with insulin or oral hypoglycemic agents, cigarette smoking, congestive heart failure, obstructive sleep apnea, active malignancy. Patients treated with weight-altering medications including tricyclic antidepressants, paroxetine, mirtazepine, lithium, valproate, gabapentin and typical and atypical antipsychotics, fluoxetine in doses greater than 20mg, bupropion, topiramate, systemic glucocorticoids and weight management drugs were also excluded. Blood samples were collected at baseline prior to initiation of weight loss therapy. Twenty lean subjects from the same genetic ancestry (Northern European White), with a BMI ≤ the 10th percentile for age and sex and no prior history of having had a BMI> 25th percentile for more than a 2 year consecutive period, were recruited from the Ottawa community. Lean subjects were excluded if they had any medical conditions affecting weight gain such as hyperthyroidism, anorexia nervosa, bulimia, major depression, or malabsorption syndromes. BMI for obese and lean subjects was categorized according to the population percentiles for age and sex using the Canadian Heart Health Survey data for subjects over the age of 18 years (data on file; Health Canada). The study protocol was approved by the Human Research Ethics Committees of the Ottawa Hospital and the University of Ottawa Heart Institute and informed consent was obtained from all participants prior to their enrolling into the program.

### Sample preparation for transcriptome analysis

2.5 ml of fasting whole blood was drawn from study subjects by standard venipuncture and directly transferred to PAXgene blood RNA tubes (Qiagen, Santa Clara, CA). PAXgene tubes were processed at designated times after phlebotomy by the PAXgene protocol. Isolation of total RNA was accomplished according to the manufacturer's instructions. Prior to further processing, RNA quality was ascertained by electropherograms on the Agilent 2100 Bioanalyzer. Extracted RNA from all samples was stored -70°C until processed for microarray hybridizations.

### Microarray hybridization and data analysis

Hybridization of 100 nanograms of labeled cRNA from each sample was carried out on Affymetrix GeneChip^® ^Human Genome U133 Plus 2.0 Arrays according to the manufacturer's instructions. Microarray data was deposited in the Gene Expression Omnibus data repository (accession number GSE18897). Gene expression signals were generated from hybridized and scanned Affymetrix arrays by the GC-RMA algorithm [54]. Probesets with a normalized average expression level of less than 50 units in all of the tested groups were eliminated from further analysis. Significance of differential gene expression was ascertained via the signal-to-noise algorithm from the GenePattern Comparative Marker Selection module [22], employing a permutation-based t-test and false discovery rate (FDR) control. The Signal-to-Noise feature selection method is a variation of the more commonly used t-test statistic and looks at the difference of the means in each of the classes scaled by the sum of the standard deviations: Sx = (μ0-μ1)/(σ0 + σ1) where μ0 is the mean of class 0 and σ0 is the standard deviation of class 0. The Signal-to-Noise statistic penalizes genes that have higher variance in each class more than those genes that have a high variance in one class and a low variance in another.

### Pathway analysis

Bioinformatic pathway analysis was conducted with the Gene Set Enrichment Analysis (GSEA) software package [27,55]. GSEA is a computational method to detect statistically significant, concordant differences in *a priori *defined gene sets (pathways) between two biological states. GSEA accomplishes this task by calculating a weighted Kolmogorov-Smirnov statistic, adjusted for gene-set size (known as the Normalized Enrichment Score, NES) for each gene-set, based on the over-representation of members of a gene-set towards the top or bottom of a list of genes ranked by the strength of their correlation (positive or negative) to one of the two phenotypes. The statistical significance of NES score is estimated by a permutation test based on random shuffling of the phenotype or tag (gene) labels. GSEA addresses the problem of multiple testing (testing hundreds of gene-sets simultaneously) by calculating a false-discovery rate and a family-wise error rate on the ES p-values.

### Quantitative real time polymerase chain reaction (RT-PCR)

Whole blood was collected in PAXgene™ blood tubes (Qiagen, Santa Clara, CA) and total RNA was extracted using the PAXgene™ blood kit. All RNA was treated with DNase I to remove genomic DNA contamination. The RNA was converted to cDNA in a 96-well microtiter plate on an ABI PRISM 7700 Sequence Detector System (Applied Biosystems, Foster City, CA) using the Applied Biosystems High Capacity cDNA archive kit. Gene expression was conducted on the Applied Biosystems 7900 using TaqMan^® ^RT-PCR technology. A global median absolute deviation (MAD) was computed from the gene expression values by taking the median deviation for each set of technical replicates, using either the Ct values or log_2 _calculated abundances. Outliers were defined as having more than five times the global MAD. Following technical and biological outlier identification the data was normalized using reference housekeeper genes. The mean Ct value of all reference genes across all samples ("global mean Ct") was subtracted from the mean Ct value of all reference genes within each sample ("sample reference mean") to determine a normalization factor for each sample. The normalization factor for a given sample was then subtracted from its Ct value resulting in a normalized Ct. All Ct values were then converted to log2 abundances.

### Class Prediction from gene expression

Class prediction (obese or lean) from gene expression data was carried out through the WEKA Explorer and WEKA Experimenter applications. First, 183 genes belonging to the 3 obese-upregulated pathways (ribosome, apoptosis and oxidative phosphorylation) were used to identify a subset of maximally informative features (genes) for classifier testing while removing irrelevant or redundant features that could negatively impact algorithm performance. Feature selection was accomplished by two independent 'filtering-based' algorithms (Information Gain and Cfs Subset Evaluator) and using 10-fold cross validation for each method [56,57]. We did not use 'wrapper-based' feature-selection because we wanted the selected features to be independent of classification algorithms [58]. Both procedures resulted in a list of genes that were then ranked based on their importance in each feature selection method. From these ranked lists, we selected a total of 11 genes that were ranked within the top 20 genes in both lists. Gene expression signals for these 11 genes were then used as input in 4 different classifiers (Naïve Bayes, Logistic Regression, Random Forests and ZeroR) representing 4 different algorithmic approaches (Bayesian, regression, decision trees and rule-based, respectively) which were independently tested for predictive performance (Additional File 13) [59,60]. Classifer-specific parameters were kept at the defaults provided in WEKA Experimenter. Each classifier used 66% of the samples for training (from a total of 34 obese plus lean subjects) and 33% for testing (chosen at random for each round) for a total of 100 iterations. For each classifier, the true positive rate, true negative rate, false positive rate, and false negative rates were calculated (average plus standard deviation over 100 iterations) and the values used to compare individual classifiers for their predictive performance.

## Competing interests

The authors SG, SG(Gorman), and JS are former or current employees of GlaxoSmithKline and have equity in the company. The other authors (RD, RM, MEH) have no disclosures.

## Authors' contributions

SG carried out the experimental design, data analysis, interpretation and drafted the manuscript. RD provided phenotype information and samples for transcriptome analysis and edited the manuscript. MEH participated in experimental design and interpretation of microarray data and manuscript editing. SG(Gorman) performed the Taqman analysis. JS coordinated the sample management, RNA isolation and microarray hybridizations. RM had overall oversight of the study and helped prepare the final version of the manuscript. All authors read and approved the final manuscript.

## Pre-publication history

The pre-publication history for this paper can be accessed here:

http://www.biomedcentral.com/1755-8794/3/56/prepub

## Supplementary Material

Additional file 1 **Comparison of expression of adipocyte and muscle specific genes in whole blood samples utilized in the current study**. Data for adipocyte-specific and muscle-specific gene expression was obtained from microarray data available on 79 different tissues from the Genomics Institute of Novartis Research Foundation http://www.gnf.org. Relative expression in whole blood was also obtained from the same source. The average expression and standard deviation in adipocyte-specific and muscle-specific gene expression observed in whole blood samples used in the current study Click here for file

Additional file 2 **PCA model output from multivariate analysis on obese and lean subjects based on whole blood gene expression signals**. Analysis of performance of the PCA model separating obese from lean subjects based on blood gene expression signals. Click here for file

Additional file 3 **Top 100 differentially expressed genes in whole blood from obese and lean subjects**. Using the GenePattern algorithm http://www.broadinstitute.org/cancer/software/genepattern/ a list of the top 50 upregulated and top 50 downregulated genes in obese and lean samples was generated and plotted on a heat-map for visualization. Higher expression levels are indicated in red and lower expression levels are indicated in blue. Genes (rows) are indicated by their Affymetrix probeset identifiers and samples (columns) are indicated by their obese or lean categories. Click here for file

Additional file 4 **Differential gene expression analysis between obese and lean subjects from blood transcriptome data**. Identification of top 200 differentially expressed genes between Obese and Lean subjects using the Comparative Marker Selection module in GenePattern. Results for top 100 upregulated and top 100 downregulated genes (Obese vs. Lean) are shown. Click here for file

Additional file 5 **Description and value ranges of the parameters used in gene-set enrichment analysis (GSEA) in the present study**. For detailed explanation of parameters and acceptable value ranges, please see additional documentation at http://www.broadinstitute.org/gsea/doc/GSEAUserGuideFrame.html. Click here for file

Additional file 6 **Custom pathway database used in the GSEA studies**. Column 1 indicates the pathway name, column 2 indicates the source for the pathway information. Subsequent columns represent the gene symbols for the genes constituting the pathway. Click here for file

Additional file 7 **List of pathways determined to be upregulated in the Obese subjects compared to the Lean subjects and vice versa**. These results were obtained by querying the pathways in the KEGG database (> = 10 and < = 200 gene members) and using the GSEA algorithm. Click here for file

Additional file 8 **List of gene members of the 3 pathways identified by GSEA as upregulated in obese subjects**. List of genes in the oxidative phosphorylation, ribosome and apoptosis signaling pathways that were expressed in blood from obese and lean subjects. The column 'Core Enrichment' describes whether a gene contributed significantly towards the enrichment of the respective pathway in the gene set enrichment analysis. Click here for file

Additional file 9 **List of pathways determined to be upregulated in the Obese subjects compared to the Lean subjects and vice versa**. These results were obtained by querying the pathways in a user-defined custom database (> = 10 and < = 200 gene members) and using the GSEA algorithm. Click here for file

Additional file 10 **Expression patterns of genes reported to be enriched in erythrocyte/reticulocyte fraction among lean and obese subjects**. The average log2 expression signal in obese and lean subjects, differential expression (log ratio) and statistical significance of the differences in expression in the two groups are indicated. Click here for file

Additional file 11 **Overexpression of erythrocyte/reticulocyte enriched genes in obese blood samples**. A scatter plot of log average expression of genes (x-axis) versus the differences of log expression between the obese and lean cohorts (y-axis) was created (also known as a MA plot). Each gene is indicated by a gray dot with the exception of genes reported to be enriched in erythrocytes/reticulocytes (compared to other blood cell types) which are shown as black pluses. A value of 0 on the y-axis signifies no differences in gene expression between the lean and obese cohorts. Click here for file

Additional file 12 **Gene set enrichment analysis between obese and lean subjects considering male only or female only cohorts**. Pathways are ranked in descending order of their enrichment for each comparison. Results with males only are shown first, followed by the results with females only. Click here for file

Additional file 13 **Gene set enrichment analysis between obese and lean subjects after scaling of gene expression data by expression levels of erythrocyte membrane protein band 2 (EMPB2) and hemoglobin D (HBD) genes respectively**. Pathways are ranked in descending order of enrichment. Top part refers to results obtained after scaling with EMPB2; bottom part shows the results following scaling with HBD. Click here for file

Additional file 14 **Description of classifiers and classifier performance evaluators used in the study**. Brief descriptions of the Naïve Bayes, Logistic Regression, Random Forests and ZeroR classifiers along with the feature selection algorithms used (Information Gain and Cfs Subset evaluator) are given. Mathematical formulas for true and false positive and negative rates (classifier evaluation metrics) are also provided. Click here for file
