# Supplementary material for: Gene expression profiling in whole blood identifies distinct biological pathways associated with obesity
Source: BMC Med Genomics. 2010 Dec 1;3:56. doi: 10.1186/1755-8794-3-56 (PMC3014865; doi:10.1186/1755-8794-3-56)
Supplement: Additional file 1 — Comparison of expression of adipocyte and muscle specific genes in whole blood samples utilized in the current study. Data for adipocyte-specific and muscle-specific gene expression was obtained from microarray data available on 79 different tissues from the Genomics Institute of Novartis Research Foundation http://www.gnf.org. Relative expression in whole blood was also obtained from the same source. The average expression and standard deviation in adipocyte-specific and muscle-specific gene expression observed in whole blood samples used in the current study [file 1755-8794-3-56-S1.DOC]

**ADDITIONAL FILE 1**

**ADDITIONAL FILE 1:** Comparison of expression of adipocyte and muscle specific genes in whole blood samples utilized in the current study.

| **ProbeID** | **Name** | **Adipocyte Expression (GNF^a^)** | **Blood Expression (GNF)** | **Average Expression (Study^b^)** |  |
| --- | --- | --- | --- | --- | --- |
| 200831_s_at | stearoyl-CoA desaturase (delta-9-desaturase) | 34443 | 118.3 | 6.29789 |  |
| 200832_s_at | stearoyl-CoA desaturase (delta-9-desaturase) | 11492.1 | 22.65 | 52.8675 |  |
| 216442_x_at | fibronectin 1 | 9514.45 | 14.6 | 2.45852 |  |
| 212464_s_at | fibronectin 1 | 9443.15 | 11.8 | 2.58345 |  |
| 212218_s_at | fatty acid synthase | 8632.2 | 40.95 | 5.21395 |  |
| 213428_s_at | collagen, type VI, alpha 1 | 8135.35 | 51.6 | 2.9365 |  |
| 208962_s_at | fatty acid desaturase 1 | 7915.75 | 11.75 | 20.2156 |  |
| 207175_at | adiponectin, C1Q and collagen domain containing | 5268.7 | 8.6 | 2.02929 |  |
| **ProbeID** | **Gene** | **Heart Expression (GNF^a^)** | **Skeletal Muscle Expression (GNF)** | **Blood Expression (GNF)** | **Average Expression (Study^b^)** |
| 203872_at | actin, alpha 1, skeletal muscle | 8438.55 | 27578.7 | 9.05 | 2.4964 |
| 204810_s_at | creatine kinase, muscle | 13424.9 | 23153.7 | 9.4 | 2.06801 |
| 209742_s_at | myosin, light polypeptide 2, regulatory, cardiac, slow | 13756.9 | 8842.55 | 4.05 | 2.21026 |
| 205766_at | titin-cap (telethonin) | 4873.6 | 6650.4 | 25.8 | 5.45479 |
| 202222_s_at | desmin | 4400.15 | 6552.8 | 6.95 | 5.34588 |
| 209904_at | troponin C, slow | 27043.6 | 5953.15 | 8.6 | 3.29465 |
| 204179_at | myoglobin | 8932 | 4359.3 | 11.95 | 2.83489 |
| 212361_s_at | ATPase, Ca++ transporting, cardiac muscle, slow twitch 2 | 1215.05 | 3636.15 | 38.9 | 68.5084 |
| 209283_at | crystallin, alpha B | 8045.45 | 2505.55 | 12.1 | 2.27635 |
| 206116_s_at | tropomyosin 1 (alpha) | 6649.45 | 2163.25 | 24.5 | 17.443 |

a: Data for adipocyte-specific and muscle-specific gene expression was obtained from microarray data available on 79 different tissues from the Genomics Institute of Novartis Research Foundation ([www.gnf.org](http://www.gnf.org/)). Relative expression in whole blood was also obtained from the same source.

b: The average expression and standard deviation in adipocyte-specific and muscle-specific gene expression observed in whole blood samples used in the current study
