# Supplementary material for: Gene expression profiling in whole blood identifies distinct biological pathways associated with obesity
Source: BMC Med Genomics. 2010 Dec 1;3:56. doi: 10.1186/1755-8794-3-56 (PMC3014865; doi:10.1186/1755-8794-3-56)
Supplement: Additional file 2 — PCA model output from multivariate analysis on obese and lean subjects based on whole blood gene expression signals. Analysis of performance of the PCA model separating obese from lean subjects based on blood gene expression signals. [file 1755-8794-3-56-S2.DOC]

**Additional File 2: PCA model output from multivariate analysis on obese and lean subjects based on whole blood gene expression signals**

| M1 - PCA_ObeseLean_RightNumProbesets_81909 | | | | | | | |  |
| --- | --- | --- | --- | --- | --- | --- | --- | --- |
| **A** | **R2X** | **R2X(cum)** | **Eigenvalue** | **Q2** | **Limit** | **Q2(cum)** | **Significance** |  |
| 0 | Cent. |  |  |  |  |  |  |  |
| 1 | 0.274 | 0.274 | 9.31 | 0.224 | 0.0295 | 0.224 | R1 |  |
| 2 | 0.143 | 0.417 | 4.85 | 0.133 | 0.0304 | 0.327 | R1 |  |
| 3 | 0.102 | 0.518 | 3.46 | 0.0883 | 0.0313 | 0.386 | R1 |  |
| 4 | 0.0622 | 0.581 | 2.12 | 0.0257 | 0.0323 | 0.402 | R2 |  |
| 5 | 0.0593 | 0.64 | 2.01 | 0.0631 | 0.0334 | 0.44 | R1 |  |
| 6 | 0.0425 | 0.682 | 1.45 | 0.0477 | 0.0346 | 0.467 | R1 |  |

Type: PCA-X Observations (N)=34, Variables (K)=12127 (X=12127, Y=0)

**Explanation of columns:**

- **A**– Index of principal components
- **R2X** - Fraction of X-variation modeled in the component
- **R2X(cum)** - Cumulative R2X up to the specified component
- **Eigenvalue** - The number of variables (K) times R2X
- **Q2** - Overall cross-validated R2X for the component
- **Limit** - Critical value of Q2 under which the component is insignificant
- **Q2(cum)** - Cumulative Q2 up to the specified component.
- **Significance** – Cross validation insignificant (NS) or significant
