# Supplementary material for: Gene expression profiling in whole blood identifies distinct biological pathways associated with obesity
Source: BMC Med Genomics. 2010 Dec 1;3:56. doi: 10.1186/1755-8794-3-56 (PMC3014865; doi:10.1186/1755-8794-3-56)
Supplement: Additional file 3 — Top 100 differentially expressed genes in whole blood from obese and lean subjects. Using the GenePattern algorithm http://www.broadinstitute.org/cancer/software/genepattern/ a list of the top 50 upregulated and top 50 downregulated genes in obese and lean samples was generated and plotted on a heat-map for visualization. Higher expression levels are indicated in red and lower expression levels are indicated in blue. Genes (rows) are indicated by their Affymetrix probeset identifiers and samples (columns) are indicated by their obese or lean categories. [file 1755-8794-3-56-S3.DOC]

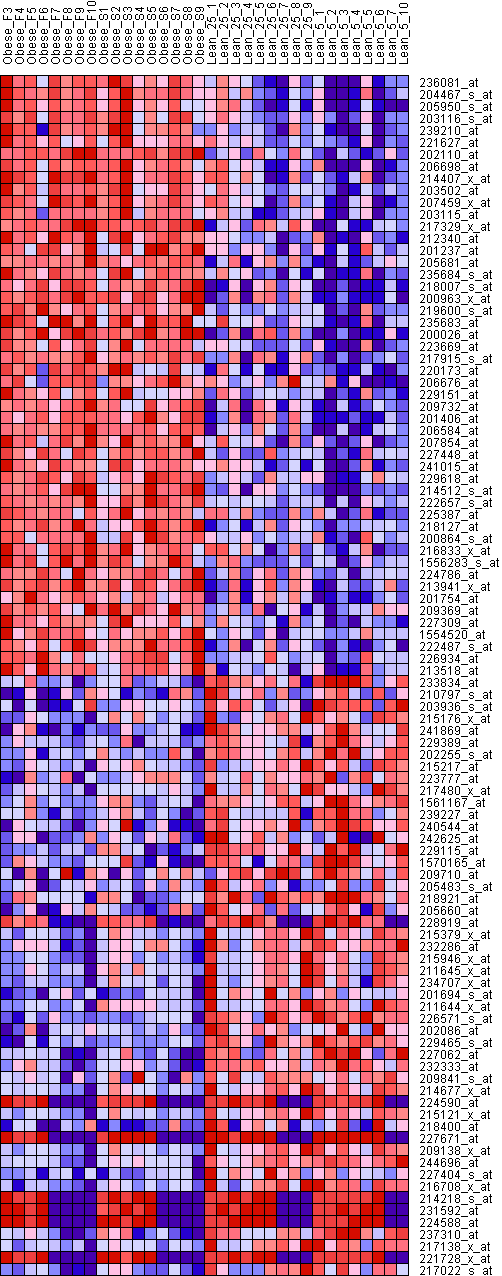
**Additional File 3: Heatmap of top 100 differentially expressed genes from blood gene expression profiling in obese and lean subjects.** Gene expression signals are color coded (shades of red indicating higher expression levels and shades of blue indicating lower expression levels). Genes are identified by Affymetrix probeset identifiers (rows) and sample types are indicated by obese or lean identifiers (columns).
