# Supplementary material for: Gene expression profiling in whole blood identifies distinct biological pathways associated with obesity
Source: BMC Med Genomics. 2010 Dec 1;3:56. doi: 10.1186/1755-8794-3-56 (PMC3014865; doi:10.1186/1755-8794-3-56)
Supplement: Additional file 5 — Description and value ranges of the parameters used in gene-set enrichment analysis (GSEA) in the present study. For detailed explanation of parameters and acceptable value ranges, please see additional documentation at http://www.broadinstitute.org/gsea/doc/GSEAUserGuideFrame.html. [file 1755-8794-3-56-S5.DOC]

**Additional File 5**

Description and value ranges of the parameters used in gene-set enrichment analysis (GSEA).

For detailed explanation of parameters and acceptable value ranges, please see additional

documentation at http://www.broadinstitute.org/gsea/doc/GSEAUserGuideFrame.html.

**Parameter Value**

**Expression Dataset**: Input dataset containing 2 files. File 1,containing gene expression signals per sample; File 2, containing sample class information (obese or lean)

**Gene Sets Database:** Pathway database queried for enrichment analysis. The KEGG pathway was used in the reported study

**Number of permutations:** 1000

**Phenotype labels:** Obese vs Lean

**Collapse dataset to gene symbols:** True

**Permutation type:** Gene-set (because of low sample numbers in each cohort, phenotype based permuations were not conducted, based on GSEA recommendations)

**Chip Platform:** HG_U133_Plus_2.chip

**Enrichment statistic:** Weighted

**Metric for ranking genes:** Signal2Noise

**Gene list sorting mode:** Real

**Gene list ordering mode:** Descending

**Max size: exclude larger sets:** 200

**Min size: exclude smaller sets:** 10

**Collapsing mode for probe sets** **=> 1 gene:** Max_probe

**Normalization mode:** Meandiv

**Randomization mode:** No_balance

**Omit features with no symbol match:** True

**Median for class metrics:** False

**Number of markers:** 100

**Plot graphs for the top sets of each phenotype:** 20

**Seed for permutation:** Timestamp

**Save random ranked lists:** False
