# Supplementary material for: Gene expression profiling in whole blood identifies distinct biological pathways associated with obesity
Source: BMC Med Genomics. 2010 Dec 1;3:56. doi: 10.1186/1755-8794-3-56 (PMC3014865; doi:10.1186/1755-8794-3-56)

**Additional File 11**

Overexpression of erythrocyte/reticulocyte enriched genes in obese blood samples. A scatter plot of log average expression of genes (x-axis) versus the differences of log expression between the obese and lean cohorts (y-axis) was created (also known as a MA plot). Each gene is indicated by a gray dot with the exception of genes reported to be enriched in erythrocytes/reticulocytes (compared to other blood cell types) which are shown as black pluses. A value of 0 on the y-axis signifies no differences in gene expression between the lean and obese cohorts. Generally, similar numbers of genes are present above and below the 0 value, implying similarities in the numbers of up- and down-regulated genes in both groups. However, a majority of the erythrocyte/reticulocyte specific genes (black pluses) show positive y-values indicating their upregulation in the obese subjects.


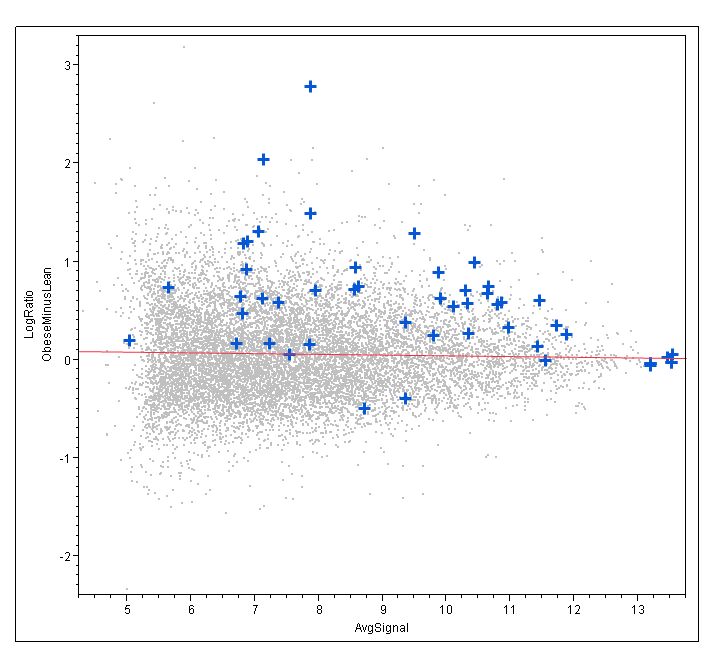

Supplement: Additional file 11 — Overexpression of erythrocyte/reticulocyte enriched genes in obese blood samples. A scatter plot of log average expression of genes (x-axis) versus the differences of log expression between the obese and lean cohorts (y-axis) was created (also known as a MA plot). Each gene is indicated by a gray dot with the exception of genes reported to be enriched in erythrocytes/reticulocytes (compared to other blood cell types) which are shown as black pluses. A value of 0 on the y-axis signifies no differences in gene expression between the lean and obese cohorts. [file 1755-8794-3-56-S11.DOC]
