# Supplementary material for: Gene expression profiling in whole blood identifies distinct biological pathways associated with obesity
Source: BMC Med Genomics. 2010 Dec 1;3:56. doi: 10.1186/1755-8794-3-56 (PMC3014865; doi:10.1186/1755-8794-3-56)
Supplement: Additional file 12 — Gene set enrichment analysis between obese and lean subjects considering male only or female only cohorts. Pathways are ranked in descending order of their enrichment for each comparison. Results with males only are shown first, followed by the results with females only. [file 1755-8794-3-56-S12.PDF]

Additional File 12

List of pathways determined to be upregulated in the Obese subjects compared to the Lean subjects and vice versa in MALE and FEMALE subjects separately. Column descriptions: **Column A**, name of KEGG pathway; **Column B**, number of genes belonging to the pathway that are found in the dataset; **Column C**, Enrichment Score ; **Column D**, Normalized enrichment score; **Column E**, permutation based p-value indicative of statistical significance of the corresponding pathway; **Column F**, a measure of the false discovery rate for the pathway; **Column G**, a measure of family-wise error rate; **Column H**, cohort that the pathway is upregulated in; **Column I,** cohort description

NAME OF KEGG PATHWAY	SIZE	ES	NES
HSA00512_O_GLYCAN_BIOSYNTHESIS	11	0.711121	1.683212
HSA00860_PORPHYRIN_AND_CHLOROPHYLL_METABOLISM	13	0.638991	1.574879
HSA04210_APOPTOSIS	55	0.451595	1.512792
HSA05010_ALZHEIMERS_DISEASE	11	0.651478	1.505134
HSA04115_P53_SIGNALING_PATHWAY	32	0.493806	1.480146
HSA04350_TGF_BETA_SIGNALING_PATHWAY	32	0.477875	1.455152
HSA00632_BENZOATE_DEGRADATION_VIA_COA_LIGATION	11	0.605947	1.41637
HSA01031_GLYCAN_STRUCTURES_BIOSYNTHESIS_2	20	0.522635	1.408715
HSA05222_SMALL_CELL_LUNG_CANCER	40	0.431787	1.357066
HSA03022_BASAL_TRANSCRIPTION_FACTORS	19	0.492486	1.346658
HSA00960_ALKALOID_BIOSYNTHESIS_II	10	0.573927	1.300439
HSA00100_BIOSYNTHESIS_OF_STEROIDS	10	0.576206	1.299517
HSA01030_GLYCAN_STRUCTURES_BIOSYNTHESIS_1	40	0.41476	1.292649
HSA02010_ABC_TRANSPORTERS_GENERAL	11	0.5361	1.270874
HSA00564_GLYCEROPHOSPHOLIPID_METABOLISM	22	0.454593	1.262476
HSA04120_UBIQUITIN_MEDIATED_PROTEOLYSIS	25	0.438822	1.261704
HSA00903_LIMONENE_AND_PINENE_DEGRADATION	13	0.506691	1.236895
HSA00190_OXIDATIVE_PHOSPHORYLATION	79	0.353228	1.233775
HSA00240_PYRIMIDINE_METABOLISM	34	0.400827	1.198826
HSA04140_REGULATION_OF_AUTOPHAGY	10	0.522893	1.193937
HSA04620_TOLL_LIKE_RECEPTOR_SIGNALING_PATHWAY	49	0.357509	1.165342
HSA04060_CYTOKINE_CYTOKINE_RECEPTOR_INTERACTION	66	0.331196	1.137732
HSA04610_COMPLEMENT_AND_COAGULATION_CASCADES	14	0.436647	1.098809
HSA00563_GLYCOSYLPHOSPHATIDYLINOSITOL_ANCHOR_BIOSYNTHES	12	0.468251	1.097365
HSA00480_GLUTATHIONE_METABOLISM	14	0.436813	1.088233
HSA01510_NEURODEGENERATIVE_DISEASES	22	0.383165	1.052583
HSA03010_RIBOSOME	54	0.312487	1.032377
HSA03050_PROTEASOME	21	0.37842	1.030163
HSA00600_SPHINGOLIPID_METABOLISM	12	0.427207	1.020743

HSA05219_BLADDER_CANCER	16	0.392691	1.017272
HSA00051_FRUCTOSE_AND_MANNOSE_METABOLISM	18	0.378483	1.01518
HSA04080_NEUROACTIVE_LIGAND_RECEPTOR_INTERACTION	27	0.349307	0.998814
HSA04370_VEGF_SIGNALING_PATHWAY	33	0.312576	0.967377
HSA04110_CELL_CYCLE	57	0.288596	0.96496
HSA04010_MAPK_SIGNALING_PATHWAY	103	0.256366	0.940174
HSA00030_PENTOSE_PHOSPHATE_PATHWAY	12	0.364039	0.88259
HSA00230_PURINE_METABOLISM	57	0.260886	0.873
HSA00450_SELENOAMINO_ACID_METABOLISM	11	0.374443	0.869346
HSA00620_PYRUVATE_METABOLISM	22	0.292003	0.813438
HSA05211_RENAL_CELL_CARCINOMA	39	0.249854	0.788188
HSA00510_N_GLYCAN_BIOSYNTHESIS	20	0.286549	0.78205
HSA04930_TYPE_II_DIABETES_MELLITUS	14	0.309938	0.778311
HSA04130_SNARE_INTERACTIONS_IN_VESICULAR_TRANSPORT	23	0.274417	0.774702
HSA00350_TYROSINE_METABOLISM	15	0.302661	0.768755
HSA05120_EPITHELIAL_CELL_SIGNALING_IN_HELICOBACTER_PYLORI_	41	0.244534	0.766374
HSA04150_MTOR_SIGNALING_PATHWAY	25	0.263748	0.750187
HSA05218_MELANOMA	22	0.267978	0.735279
HSA00251_GLUTAMATE_METABOLISM	16	0.285532	0.722122
HSA00220_UREA_CYCLE_AND_METABOLISM_OF_AMINO_GROUPS	10	0.2842	0.651384
HSA04320_DORSO_VENTRAL_AXIS_FORMATION	13	0.254016	0.625542
HSA04360_AXON_GUIDANCE	40	0.196838	0.612102
HSA04514_CELL_ADHESION_MOLECULES	47	-0.50184	-1.89289
HSA00970_AMINOACYL_TRNA_BIOSYNTHESIS	20	-0.60265	-1.78568
HSA04660_T_CELL_RECEPTOR_SIGNALING_PATHWAY	55	-0.44912	-1.72747
HSA05221_ACUTE_MYELOID_LEUKEMIA	30	-0.49103	-1.67379
HSA04640_HEMATOPOIETIC_CELL_LINEAGE	44	-0.42286	-1.56392
HSA04070_PHOSPHATIDYLINOSITOL_SIGNALING_SYSTEM	38	-0.43707	-1.55201
HSA00640_PROPANOATE_METABOLISM	18	-0.52154	-1.53292
HSA00340_HISTIDINE_METABOLISM	15	-0.54342	-1.52492
HSA04020_CALCIUM_SIGNALING_PATHWAY	50	-0.39699	-1.52145
HSA04612_ANTIGEN_PROCESSING_AND_PRESENTATION	43	-0.39889	-1.51713
HSA04940_TYPE_I_DIABETES_MELLITUS	25	-0.45521	-1.49179
HSA00410_BETA_ALANINE_METABOLISM	11	-0.55931	-1.43325
HSA04662_B_CELL_RECEPTOR_SIGNALING_PATHWAY	39	-0.37083	-1.36568
HSA04540_GAP_JUNCTION	31	-0.37985	-1.32167
HSA00380_TRYPTOPHAN_METABOLISM	23	-0.41295	-1.31173
HSA00310_LYSINE_DEGRADATION	20	-0.4306	-1.30549
HSA00561_GLYCEROLIPID_METABOLISM	17	-0.43634	-1.27761
HSA04740_OLFACTORY_TRANSDUCTION	10	-0.49755	-1.25563
HSA04810_REGULATION_OF_ACTIN_CYTOSKELETON	94	-0.26461	-1.16584
HSA04916_MELANOGENESIS	34	-0.3341	-1.14717
HSA04510_FOCAL_ADHESION	81	-0.26854	-1.12632
HSA00071_FATTY_ACID_METABOLISM	20	-0.36431	-1.11363
HSA00930_CAPROLACTAM_DEGRADATION	11	-0.43045	-1.10481

HSA04730_LONG_TERM_DEPRESSION	24	-0.33915	-1.08212
HSA04912_GNRH_SIGNALING_PATHWAY	38	-0.29807	-1.07868
HSA00562_INOSITOL_PHOSPHATE_METABOLISM	22	-0.3414	-1.06301
HSA00252_ALANINE_AND_ASPARTATE_METABOLISM	11	-0.41272	-1.05223
HSA04012_ERBB_SIGNALING_PATHWAY	43	-0.28686	-1.04973
HSA00120_BILE_ACID_BIOSYNTHESIS	15	-0.37529	-1.03315
HSA03320_PPAR_SIGNALING_PATHWAY	19	-0.34482	-1.03167
HSA05220_CHRONIC_MYELOID_LEUKEMIA	46	-0.27532	-1.02722
HSA00010_GLYCOLYSIS_AND_GLUCONEOGENESIS	30	-0.30227	-1.01998
HSA04664_FC_EPSILON_RI_SIGNALING_PATHWAY	39	-0.28142	-0.99945
HSA04512_ECM_RECEPTOR_INTERACTION	12	-0.36917	-0.98867
HSA00500_STARCH_AND_SUCROSE_METABOLISM	24	-0.30926	-0.98728
HSA00020_CITRATE_CYCLE	18	-0.33212	-0.98401
HSA04650_NATURAL_KILLER_CELL_MEDIATED_CYTOTOXICITY	72	-0.23811	-0.96977
HSA00280_VALINE_LEUCINE_AND_ISOLEUCINE_DEGRADATION	22	-0.31912	-0.96221
HSA05214_GLIOMA	33	-0.2696	-0.9432
HSA05217_BASAL_CELL_CARCINOMA	12	-0.35629	-0.94208
HSA04340_HEDGEHOG_SIGNALING_PATHWAY	11	-0.34741	-0.9054
HSA04530_TIGHT_JUNCTION	46	-0.23276	-0.88256
HSA04330_NOTCH_SIGNALING_PATHWAY	27	-0.26412	-0.86614
HSA00650_BUTANOATE_METABOLISM	21	-0.28663	-0.86204
HSA04310_WNT_SIGNALING_PATHWAY	67	-0.21301	-0.8576
HSA00710_CARBON_FIXATION	10	-0.34351	-0.84744
HSA05110_CHOLERA_INFECTION	25	-0.24913	-0.81246
HSA05131_PATHOGENIC_ESCHERICHIA_COLI_INFECTION_EPEC	29	-0.23333	-0.80139
HSA05215_PROSTATE_CANCER	46	-0.21108	-0.79595
HSA04920_ADIPOCYTOKINE_SIGNALING_PATHWAY	38	-0.22144	-0.78746
HSA05130_PATHOGENIC_ESCHERICHIA_COLI_INFECTION_EHEC	29	-0.23333	-0.78561
HSA04670_LEUKOCYTE_TRANSENDOTHELIAL_MIGRATION	55	-0.19837	-0.7794
HSA00790_FOLATE_BIOSYNTHESIS	13	-0.28553	-0.76368
HSA04720_LONG_TERM_POTENTIATION	37	-0.2103	-0.75715
HSA00052_GALACTOSE_METABOLISM	12	-0.28574	-0.75246
HSA05210_COLORECTAL_CANCER	42	-0.20552	-0.7523
HSA04520_ADHERENS_JUNCTION	39	-0.20717	-0.74602
HSA00260_GLYCINE_SERINE_AND_THREONINE_METABOLISM	13	-0.26749	-0.739
HSA01032_GLYCAN_STRUCTURES_DEGRADATION	13	-0.27179	-0.73666
HSA05223_NON_SMALL_CELL_LUNG_CANCER	29	-0.21597	-0.72357
HSA03020_RNA_POLYMERASE	13	-0.25197	-0.69447
HSA04630_JAK_STAT_SIGNALING_PATHWAY	58	-0.17042	-0.67515
HSA05213_ENDOMETRIAL_CANCER	29	-0.19867	-0.66858
HSA04910_INSULIN_SIGNALING_PATHWAY	65	-0.15555	-0.6365
HSA05216_THYROID_CANCER	17	-0.21543	-0.63114
HSA05030_AMYOTROPHIC_LATERAL_SCLEROSIS	12	-0.23189	-0.60851
HSA00530_AMINOSUGARS_METABOLISM	15	-0.20515	-0.58211
HSA05212_PANCREATIC_CANCER	41	-0.14368	-0.51805

HSA05040_HUNTINGTONS_DISEASE	17	-0.17591	-0.50326
HSA03010_RIBOSOME	54	0.536861	1.747148
HSA00512_O_GLYCAN_BIOSYNTHESIS	11	0.69109	1.641252
HSA04350_TGF_BETA_SIGNALING_PATHWAY	32	0.493733	1.479835
HSA00632_BENZOATE_DEGRADATION_VIA_COA_LIGATION	11	0.630888	1.47193
HSA03022_BASAL_TRANSCRIPTION_FACTORS	19	0.545972	1.457375
HSA04115_P53_SIGNALING_PATHWAY	32	0.487791	1.453138
HSA00860_PORPHYRIN_AND_CHLOROPHYLL_METABOLISM	13	0.586131	1.431356
HSA04210_APOPTOSIS	55	0.421836	1.353844
HSA05222_SMALL_CELL_LUNG_CANCER	40	0.412613	1.295047
HSA00564_GLYCEROPHOSPHOLIPID_METABOLISM	22	0.464976	1.289547
HSA00100_BIOSYNTHESIS_OF_STEROIDS	10	0.567855	1.273755
HSA00190_OXIDATIVE_PHOSPHORYLATION	79	0.365813	1.266774
HSA01031_GLYCAN_STRUCTURES_BIOSYNTHESIS_2	20	0.453802	1.215419
HSA00960_ALKALOID_BIOSYNTHESIS_II	10	0.540402	1.211178
HSA00563_GLYCOSYLPHOSPHATIDYLINOSITOL_ANCHOR_BIOSYNTHES	12	0.508917	1.206607
HSA01030_GLYCAN_STRUCTURES_BIOSYNTHESIS_1	40	0.383815	1.180858
HSA04110_CELL_CYCLE	57	0.341378	1.129906
HSA00051_FRUCTOSE_AND_MANNOSE_METABOLISM	18	0.409829	1.077833
HSA04120_UBIQUITIN_MEDIATED_PROTEOLYSIS	25	0.381184	1.063515
HSA00650_BUTANOATE_METABOLISM	21	0.377235	1.01768
HSA02010_ABC_TRANSPORTERS_GENERAL	11	0.428868	1.013316
HSA00620_PYRUVATE_METABOLISM	22	0.3673	1.006406
HSA00903_LIMONENE_AND_PINENE_DEGRADATION	13	0.4198	1.005703
HSA00251_GLUTAMATE_METABOLISM	16	0.363567	0.936981
HSA00510_N_GLYCAN_BIOSYNTHESIS	20	0.351076	0.924998
HSA04080_NEUROACTIVE_LIGAND_RECEPTOR_INTERACTION	27	0.320721	0.923959
HSA00260_GLYCINE_SERINE_AND_THREONINE_METABOLISM	13	0.375429	0.912676
HSA05130_PATHOGENIC_ESCHERICHIA_COLI_INFECTION_EHEC	29	0.310623	0.908852
HSA04130_SNARE_INTERACTIONS_IN_VESICULAR_TRANSPORT	23	0.325076	0.90503
HSA05131_PATHOGENIC_ESCHERICHIA_COLI_INFECTION_EPEC	29	0.310623	0.891238
HSA04060_CYTOKINE_CYTOKINE_RECEPTOR_INTERACTION	66	0.26208	0.868896
HSA04140_REGULATION_OF_AUTOPHAGY	10	0.379385	0.857252
HSA04512_ECM_RECEPTOR_INTERACTION	12	0.362459	0.849075
HSA00310_LYSINE_DEGRADATION	20	0.312271	0.843987
HSA03050_PROTEASOME	21	0.305459	0.824108
HSA05010_ALZHEIMERS_DISEASE	11	0.352308	0.820904
HSA05218_MELANOMA	22	0.292294	0.788699
HSA00500_STARCH_AND_SUCROSE_METABOLISM	24	0.27176	0.768115
HSA00240_PYRIMIDINE_METABOLISM	34	0.246126	0.736026
HSA00790_FOLATE_BIOSYNTHESIS	13	0.29808	0.720765
HSA00052_GALACTOSE_METABOLISM	12	0.288759	0.675534
HSA00450_SELENOAMINO_ACID_METABOLISM	11	0.283435	0.66424
HSA00071_FATTY_ACID_METABOLISM	20	0.246165	0.654546
HSA04620_TOLL_LIKE_RECEPTOR_SIGNALING_PATHWAY	49	0.20688	0.65359

HSA00230_PURINE_METABOLISM	57	0.197127	0.649361
HSA05211_RENAL_CELL_CARCINOMA	39	0.209123	0.640896
HSA00600_SPHINGOLIPID_METABOLISM	12	0.247705	0.593325
HSA04920_ADIPOCYTOKINE_SIGNALING_PATHWAY	38	0.177947	0.539314
HSA05212_PANCREATIC_CANCER	41	0.153426	0.477618
HSA01510_NEURODEGENERATIVE_DISEASES	22	0.172756	0.471719
HSA00410_BETA_ALANINE_METABOLISM	11	-0.71106	-1.89478
HSA04940_TYPE_I_DIABETES_MELLITUS	25	-0.51485	-1.77426
HSA04612_ANTIGEN_PROCESSING_AND_PRESENTATION	43	-0.46313	-1.74471
HSA04514_CELL_ADHESION_MOLECULES	47	-0.45535	-1.73051
HSA05120_EPITHELIAL_CELL_SIGNALING_IN_HELICOBACTER_PYLORI_	41	-0.44586	-1.68822
HSA04640_HEMATOPOIETIC_CELL_LINEAGE	44	-0.41085	-1.55626
HSA00340_HISTIDINE_METABOLISM	15	-0.52196	-1.52633
HSA00010_GLYCOLYSIS_AND_GLUCONEOGENESIS	30	-0.43172	-1.46134
HSA04020_CALCIUM_SIGNALING_PATHWAY	50	-0.35773	-1.41143
HSA04916_MELANOGENESIS	34	-0.38253	-1.38722
HSA04912_GNRH_SIGNALING_PATHWAY	38	-0.37463	-1.36769
HSA01032_GLYCAN_STRUCTURES_DEGRADATION	13	-0.46862	-1.3444
HSA04540_GAP_JUNCTION	31	-0.36694	-1.29245
HSA00710_CARBON_FIXATION	10	-0.48991	-1.28889
HSA04330_NOTCH_SIGNALING_PATHWAY	27	-0.38115	-1.28644
HSA04810_REGULATION_OF_ACTIN_CYTOSKELETON	94	-0.28835	-1.26481
HSA04510_FOCAL_ADHESION	81	-0.28945	-1.25231
HSA05221_ACUTE_MYELOID_LEUKEMIA	30	-0.35866	-1.24496
HSA04664_FC_EPSILON_RI_SIGNALING_PATHWAY	39	-0.33419	-1.21847
HSA00562_INOSITOL_PHOSPHATE_METABOLISM	22	-0.36793	-1.21022
HSA04670_LEUKOCYTE_TRANSENDOTHELIAL_MIGRATION	55	-0.30026	-1.20077
HSA00561_GLYCEROLIPID_METABOLISM	17	-0.39818	-1.19416
HSA00020_CITRATE_CYCLE	18	-0.37687	-1.14775
HSA04650_NATURAL_KILLER_CELL_MEDIATED_CYTOTOXICITY	72	-0.27629	-1.14131
HSA00970_AMINOACYL_TRNA_BIOSYNTHESIS	20	-0.35076	-1.11402
HSA05219_BLADDER_CANCER	16	-0.37433	-1.11082
HSA00640_PROPANOATE_METABOLISM	18	-0.36046	-1.11014
HSA05215_PROSTATE_CANCER	46	-0.28365	-1.08876
HSA04740_OLFACTORY_TRANSDUCTION	10	-0.41692	-1.08247
HSA04010_MAPK_SIGNALING_PATHWAY	103	-0.23712	-1.07497
HSA04662_B_CELL_RECEPTOR_SIGNALING_PATHWAY	39	-0.28668	-1.06842
HSA00380_TRYPTOPHAN_METABOLISM	23	-0.31983	-1.05951
HSA04910_INSULIN_SIGNALING_PATHWAY	65	-0.25466	-1.05495
HSA05110_CHOLERA_INFECTION	25	-0.31465	-1.05127
HSA00280_VALINE_LEUCINE_AND_ISOLEUCINE_DEGRADATION	22	-0.31211	-1.02183
HSA04530_TIGHT_JUNCTION	46	-0.26109	-1.01403
HSA04370_VEGF_SIGNALING_PATHWAY	33	-0.2771	-1.00287
HSA00030_PENTOSE_PHOSPHATE_PATHWAY	12	-0.36805	-1.00085
HSA00930_CAPROLACTAM_DEGRADATION	11	-0.37283	-0.99266

HSA04730_LONG_TERM_DEPRESSION	24	-0.29508	-0.98237
HSA04340_HEDGEHOG_SIGNALING_PATHWAY	11	-0.35524	-0.964
HSA00480_GLUTATHIONE_METABOLISM	14	-0.33344	-0.96337
HSA04630_JAK_STAT_SIGNALING_PATHWAY	58	-0.23427	-0.95502
HSA04070_PHOSPHATIDYLINOSITOL_SIGNALING_SYSTEM	38	-0.2506	-0.94137
HSA04660_T_CELL_RECEPTOR_SIGNALING_PATHWAY	55	-0.22766	-0.93184
HSA04310_WNT_SIGNALING_PATHWAY	67	-0.22755	-0.92795
HSA00530_AMINOSUGARS_METABOLISM	15	-0.32249	-0.92587
HSA00120_BILE_ACID_BIOSYNTHESIS	15	-0.31427	-0.922
HSA04360_AXON_GUIDANCE	40	-0.24278	-0.9073
HSA04012_ERBB_SIGNALING_PATHWAY	43	-0.24403	-0.9046
HSA05213_ENDOMETRIAL_CANCER	29	-0.25989	-0.89987
HSA05040_HUNTINGTONS_DISEASE	17	-0.30557	-0.8994
HSA04610_COMPLEMENT_AND_COAGULATION_CASCADES	14	-0.3154	-0.89228
HSA03320_PPAR_SIGNALING_PATHWAY	19	-0.28398	-0.8874
HSA05210_COLORECTAL_CANCER	42	-0.23254	-0.87193
HSA05216_THYROID_CANCER	17	-0.27707	-0.85652
HSA04520_ADHERENS_JUNCTION	39	-0.22144	-0.84777
HSA05217_BASAL_CELL_CARCINOMA	12	-0.30688	-0.84028
HSA00350_TYROSINE_METABOLISM	15	-0.28481	-0.81281
HSA04320_DORSO_VENTRAL_AXIS_FORMATION	13	-0.28911	-0.80114
HSA05030_AMYOTROPHIC_LATERAL_SCLEROSIS	12	-0.2905	-0.79146
HSA00252_ALANINE_AND_ASPARTATE_METABOLISM	11	-0.29273	-0.77638
HSA04930_TYPE_II_DIABETES_MELLITUS	14	-0.26283	-0.72437
HSA04720_LONG_TERM_POTENTIATION	37	-0.19405	-0.71367
HSA05223_NON_SMALL_CELL_LUNG_CANCER	29	-0.21121	-0.70499
HSA05214_GLIOMA	33	-0.19473	-0.70124
HSA03020_RNA_POLYMERASE	13	-0.24518	-0.69161
HSA00220_UREA_CYCLE_AND_METABOLISM_OF_AMINO_GROUPS	10	-0.2656	-0.67403
HSA05220_CHRONIC_MYELOID_LEUKEMIA	46	-0.16286	-0.61053
HSA04150_MTOR_SIGNALING_PATHWAY	25	-0.1524	-0.50274

NOM p-va	FDR q-va	FWER p-v	Upregula	Cohort
0.003221	0.319931	0.408	Obese	Males only
0.038143	0.486906	0.807	Obese	Males only
0.019405	0.553513	0.945	Obese	Males only
0.043269	0.441366	0.951	Obese	Males only
0.036474	0.428976	0.976	Obese	Males only
0.039271	0.427337	0.989	Obese	Males only
0.099668	0.478825	0.996	Obese	Males only
0.064319	0.440853	0.998	Obese	Males only
0.084286	0.545854	0.999	Obese	Males only
0.106984	0.52365	1	Obese	Males only
0.13253	0.622396	1	Obese	Males only
0.168053	0.572741	1	Obese	Males only
0.127072	0.547623	1	Obese	Males only
0.168285	0.571005	1	Obese	Males only
0.171946	0.554813	1	Obese	Males only
0.154519	0.522801	1	Obese	Males only
0.213355	0.557079	1	Obese	Males only
0.136842	0.534464	1	Obese	Males only
0.210219	0.595516	1	Obese	Males only
0.267974	0.578707	1	Obese	Males only
0.216643	0.621771	1	Obese	Males only
0.264668	0.665149	1	Obese	Males only
0.364821	0.739676	1	Obese	Males only
0.363176	0.712879	1	Obese	Males only
0.333871	0.70896	1	Obese	Males only
0.398724	0.770207	1	Obese	Males only
0.41337	0.792904	1	Obese	Males only
0.422556	0.770102	1	Obese	Males only
0.458529	0.767003	1	Obese	Males only

0.446541	0.749773	1	Obese	Males only
0.446875	0.730205	1	Obese	Males only
0.452941	0.744198	1	Obese	Males only
0.490141	0.792471	1	Obese	Males only
0.52412	0.774044	1	Obese	Males only
0.590571	0.806256	1	Obese	Males only
0.614876	0.909393	1	Obese	Males only
0.678036	0.904345	1	Obese	Males only
0.614498	0.88797	1	Obese	Males only
0.747706	0.973689	1	Obese	Males only
0.784448	0.995158	1	Obese	Males only
0.771723	0.981524	1	Obese	Males only
0.746933	0.964063	1	Obese	Males only
0.757296	0.947572	1	Obese	Males only
0.772358	0.934772	1	Obese	Males only
0.840845	0.917286	1	Obese	Males only
0.805723	0.920285	1	Obese	Males only
0.844828	0.920449	1	Obese	Males only
0.828704	0.916307	1	Obese	Males only
0.887622	0.968027	1	Obese	Males only
0.9264	0.966601	1	Obese	Males only
0.966667	0.955226	1	Obese	Males only
0	0.13949	0.079	Lean	Males only
0.005634	0.201333	0.207	Lean	Males only
0.003922	0.204645	0.301	Lean	Males only
0.015432	0.225	0.409	Lean	Males only
0.014706	0.397427	0.689	Lean	Males only
0.019802	0.355543	0.716	Lean	Males only
0.031339	0.343742	0.759	Lean	Males only
0.036827	0.312647	0.77	Lean	Males only
0.003817	0.283007	0.774	Lean	Males only
0.028881	0.263076	0.786	Lean	Males only
0.052023	0.282816	0.821	Lean	Males only
0.102703	0.368535	0.914	Lean	Males only
0.089457	0.49455	0.972	Lean	Males only
0.124579	0.577579	0.99	Lean	Males only
0.136943	0.56481	0.993	Lean	Males only
0.116071	0.544943	0.993	Lean	Males only
0.157754	0.582651	0.998	Lean	Males only
0.205446	0.61287	0.999	Lean	Males only
0.126263	0.865576	1	Lean	Males only
0.238854	0.884216	1	Lean	Males only
0.236842	0.917571	1	Lean	Males only
0.256198	0.921821	1	Lean	Males only
0.31401	0.913579	1	Lean	Males only

0.344411	0.953727	1	Lean	Males only
0.29582	0.926708	1	Lean	Males only
0.346154	0.946307	1	Lean	Males only
0.393782	0.948357	1	Lean	Males only
0.360856	0.922907	1	Lean	Males only
0.40404	0.945652	1	Lean	Males only
0.386921	0.919024	1	Lean	Males only
0.435714	0.903766	1	Lean	Males only
0.421384	0.897039	1	Lean	Males only
0.464883	0.935108	1	Lean	Males only
0.479784	0.94208	1	Lean	Males only
0.45082	0.919574	1	Lean	Males only
0.457995	0.904606	1	Lean	Males only
0.536481	0.925629	1	Lean	Males only
0.5	0.922786	1	Lean	Males only
0.554817	0.954892	1	Lean	Males only
0.552083	0.933696	1	Lean	Males only
0.557333	1	1	Lean	Males only
0.730627	1	1	Lean	Males only
0.675325	1	1	Lean	Males only
0.682353	1	1	Lean	Males only
0.769231	1	1	Lean	Males only
0.643979	1	1	Lean	Males only
0.753799	1	1	Lean	Males only
0.830189	1	1	Lean	Males only
0.832714	1	1	Lean	Males only
0.819444	1	1	Lean	Males only
0.807818	1	1	Lean	Males only
0.907063	1	1	Lean	Males only
0.756627	1	1	Lean	Males only
0.86	1	1	Lean	Males only
0.800487	1	1	Lean	Males only
0.888889	1	1	Lean	Males only
0.890729	1	1	Lean	Males only
0.806916	1	1	Lean	Males only
0.811828	1	1	Lean	Males only
0.897516	1	1	Lean	Males only
0.846995	1	1	Lean	Males only
0.975207	1	1	Lean	Males only
0.926554	1	1	Lean	Males only
0.995652	1	1	Lean	Males only
0.926554	1	1	Lean	Males only
0.94132	1	1	Lean	Males only
0.955224	1	1	Lean	Males only
1	1	1	Lean	Males only

0.99726	0.992982	1	Lean	Males only
0.001263	0.108663	0.172	Obese	Females only
0.012365	0.210829	0.527	Obese	Females only
0.046512	0.641683	0.968	Obese	Females only
0.051563	0.513645	0.975	Obese	Females only
0.058405	0.46021	0.983	Obese	Females only
0.036096	0.396516	0.986	Obese	Females only
0.07465	0.396111	0.994	Obese	Females only
0.072061	0.59521	1	Obese	Females only
0.122807	0.754234	1	Obese	Females only
0.156727	0.700565	1	Obese	Females only
0.15969	0.693274	1	Obese	Females only
0.10453	0.66117	1	Obese	Females only
0.218796	0.786638	1	Obese	Females only
0.232964	0.744721	1	Obese	Females only
0.247649	0.70911	1	Obese	Females only
0.193591	0.754454	1	Obese	Females only
0.281407	0.879058	1	Obese	Females only
0.368046	1	1	Obese	Females only
0.388363	1	1	Obese	Females only
0.451567	1	1	Obese	Females only
0.446677	1	1	Obese	Females only
0.451009	1	1	Obese	Females only
0.466165	1	1	Obese	Females only
0.571429	1	1	Obese	Females only
0.585091	1	1	Obese	Females only
0.586255	1	1	Obese	Females only
0.595202	1	1	Obese	Females only
0.630936	1	1	Obese	Females only
0.599729	1	1	Obese	Females only
0.648684	1	1	Obese	Females only
0.686642	1	1	Obese	Females only
0.663492	1	1	Obese	Females only
0.685714	1	1	Obese	Females only
0.698413	1	1	Obese	Females only
0.70564	1	1	Obese	Females only
0.714063	1	1	Obese	Females only
0.777624	1	1	Obese	Females only
0.785511	1	1	Obese	Females only
0.846575	1	1	Obese	Females only
0.850539	1	1	Obese	Females only
0.888025	1	1	Obese	Females only
0.901235	1	1	Obese	Females only
0.908322	1	1	Obese	Females only
0.945736	1	1	Obese	Females only

0.964504	1	1	Obese	Females only
0.956349	1	1	Obese	Females only
0.944858	1	1	Obese	Females only
0.994595	1	1	Obese	Females only
0.998684	1	1	Obese	Females only
0.991632	0.99494	1	Obese	Females only
0.002653	0.147772	0.069	Lean	Females only
0.003584	0.20717	0.182	Lean	Females only
0	0.175182	0.229	Lean	Females only
0	0.145873	0.25	Lean	Females only
0.004425	0.170461	0.344	Lean	Females only
0.013216	0.354662	0.649	Lean	Females only
0.036517	0.366336	0.715	Lean	Females only
0.055147	0.466602	0.832	Lean	Females only
0.048	0.559707	0.915	Lean	Females only
0.074074	0.572223	0.946	Lean	Females only
0.068807	0.576446	0.964	Lean	Females only
0.143293	0.596061	0.974	Lean	Females only
0.121771	0.701494	0.989	Lean	Females only
0.185393	0.662466	0.99	Lean	Females only
0.142322	0.625916	0.99	Lean	Females only
0.097015	0.649731	0.992	Lean	Females only
0.095541	0.648146	0.994	Lean	Females only
0.107759	0.637132	0.996	Lean	Females only
0.149533	0.684717	0.997	Lean	Females only
0.177083	0.676249	0.997	Lean	Females only
0.155963	0.669424	0.997	Lean	Females only
0.213115	0.656078	0.998	Lean	Females only
0.270517	0.765426	1	Lean	Females only
0.201258	0.753143	1	Lean	Females only
0.265517	0.812509	1	Lean	Females only
0.309598	0.79063	1	Lean	Females only
0.282051	0.763512	1	Lean	Females only
0.292793	0.805308	1	Lean	Females only
0.344538	0.796256	1	Lean	Females only
0.280303	0.791706	1	Lean	Females only
0.318584	0.788416	1	Lean	Females only
0.345763	0.78806	1	Lean	Females only
0.342857	0.777808	1	Lean	Females only
0.367589	0.766675	1	Lean	Females only
0.427046	0.828425	1	Lean	Females only
0.401042	0.82621	1	Lean	Females only
0.43913	0.83798	1	Lean	Females only
0.427673	0.82271	1	Lean	Females only
0.474667	0.824636	1	Lean	Females only

0.469751	0.832923	1	Lean	Females only
0.498567	0.864778	1	Lean	Females only
0.510703	0.846143	1	Lean	Females only
0.565657	0.848271	1	Lean	Females only
0.532468	0.865639	1	Lean	Females only
0.612245	0.87335	1	Lean	Females only
0.627551	0.863848	1	Lean	Females only
0.557229	0.851233	1	Lean	Females only
0.535604	0.843312	1	Lean	Females only
0.622881	0.86329	1	Lean	Females only
0.632558	0.852804	1	Lean	Females only
0.606742	0.847544	1	Lean	Females only
0.56869	0.832413	1	Lean	Females only
0.592593	0.832768	1	Lean	Females only
0.647059	0.828179	1	Lean	Females only
0.711382	0.846129	1	Lean	Females only
0.676191	0.862976	1	Lean	Females only
0.771028	0.865966	1	Lean	Females only
0.666667	0.866973	1	Lean	Females only
0.693291	0.903756	1	Lean	Females only
0.760116	0.908188	1	Lean	Females only
0.756173	0.909471	1	Lean	Females only
0.761773	0.916733	1	Lean	Females only
0.850153	0.97073	1	Lean	Females only
0.925	0.968801	1	Lean	Females only
0.906504	0.9628	1	Lean	Females only
0.949807	0.952344	1	Lean	Females only
0.839879	0.94738	1	Lean	Females only
0.871935	0.94799	1	Lean	Females only
1	0.975088	1	Lean	Females only
0.996504	0.993238	1	Lean	Females only
