# Supplementary material for: Gene expression profiling in whole blood identifies distinct biological pathways associated with obesity
Source: BMC Med Genomics. 2010 Dec 1;3:56. doi: 10.1186/1755-8794-3-56 (PMC3014865; doi:10.1186/1755-8794-3-56)
Supplement: Additional file 13 — Gene set enrichment analysis between obese and lean subjects after scaling of gene expression data by expression levels of erythrocyte membrane protein band 2 (EMPB2) and hemoglobin D (HBD) genes respectively. Pathways are ranked in descending order of enrichment. Top part refers to results obtained after scaling with EMPB2; bottom part shows the results following scaling with HBD. [file 1755-8794-3-56-S13.PDF]

Additional File 13

List of pathways determined to be upregulated in the Obese subjects compared to the Lean subjects and vice versa after adjustment of gene expression signals with EMPB2 and HemoglobinD signals. Column descriptions: **Column A**, name of KEGG pathway; **Column B**, number of genes belonging to the pathway that are found in the dataset; **Column C**, Enrichment Score ; **Column D**, Normalized enrichment score; **Column E**, permutation based p-value indicative of statistical significance of the corresponding pathway; **Column F**, a measure of the false discovery rate for the pathway; **Column G**, a measure of family-wise error rate; **Column H**, cohort that the pathway is upregulated in; **Column I,** cohort description

NAME OF KEGG PATHWAY	SIZE	ES
HSA03010_RIBOSOME	54	0.287479
HSA03022_BASAL_TRANSCRIPTION_FACTORS	19	0.354933
HSA04350_TGF_BETA_SIGNALING_PATHWAY	32	0.135674
HSA00100_BIOSYNTHESIS_OF_STEROIDS	10	0.188938
HSA04810_REGULATION_OF_ACTIN_CYTOSKELETON	94	-0.36859
HSA04612_ANTIGEN_PROCESSING_AND_PRESENTATION	43	-0.40629
HSA05219_BLADDER_CANCER	16	-0.50882
HSA04360_AXON_GUIDANCE	40	-0.3975
HSA04650_NATURAL_KILLER_CELL_MEDIATED_CYTOTOXICITY	72	-0.3656
HSA05221_ACUTE_MYELOID_LEUKEMIA	30	-0.42668
HSA04540_GAP_JUNCTION	31	-0.41063
HSA00710_CARBON_FIXATION	10	-0.56881
HSA05120_EPITHELIAL_CELL_SIGNALING_IN_HELICOBACTER_PYLORI_INFECTION	41	-0.38262
HSA00030_PENTOSE_PHOSPHATE_PATHWAY	12	-0.51806
HSA05223_NON_SMALL_CELL_LUNG_CANCER	29	-0.40348
HSA05214_GLIOMA	33	-0.38515
HSA04940_TYPE_I_DIABETES_MELLITUS	25	-0.41599
HSA04370_VEGF_SIGNALING_PATHWAY	33	-0.37896
HSA04510_FOCAL_ADHESION	81	-0.31747
HSA04012_ERBB_SIGNALING_PATHWAY	43	-0.35022
HSA00340_HISTIDINE_METABOLISM	15	-0.45134
HSA04070_PHOSPHATIDYLINOSITOL_SIGNALING_SYSTEM	38	-0.34818
HSA04664_FC_EPSILON_RI_SIGNALING_PATHWAY	39	-0.34661
HSA04514_CELL_ADHESION_MOLECULES	47	-0.33198
HSA01030_GLYCAN_STRUCTURES_BIOSYNTHESIS_1	40	-0.33721
HSA00010_GLYCOLYSIS_AND_GLUCONEOGENESIS	30	-0.36181
HSA00380_TRYPTOPHAN_METABOLISM	23	-0.37802
HSA05220_CHRONIC_MYELOID_LEUKEMIA	46	-0.32726
HSA04010_MAPK_SIGNALING_PATHWAY	103	-0.28647
HSA04662_B_CELL_RECEPTOR_SIGNALING_PATHWAY	39	-0.33934

HSA04730_LONG_TERM_DEPRESSION	24	-0.37368
HSA00350_TYROSINE_METABOLISM	15	-0.41752
HSA04330_NOTCH_SIGNALING_PATHWAY	27	-0.36345
HSA04020_CALCIUM_SIGNALING_PATHWAY	50	-0.31352
HSA04630_JAK_STAT_SIGNALING_PATHWAY	58	-0.30614
HSA00970_AMINOACYL_TRNA_BIOSYNTHESIS	20	-0.37563
HSA00510_N_GLYCAN_BIOSYNTHESIS	20	-0.38463
HSA05030_AMYOTROPHIC_LATERAL_SCLEROSIS	12	-0.43445
HSA00450_SELENOAMINO_ACID_METABOLISM	11	-0.44814
HSA05211_RENAL_CELL_CARCINOMA	39	-0.3142
HSA01510_NEURODEGENERATIVE_DISEASES	22	-0.36397
HSA04310_WNT_SIGNALING_PATHWAY	67	-0.28826
HSA00640_PROPANOATE_METABOLISM	18	-0.37751
HSA05010_ALZHEIMERS_DISEASE	11	-0.43908
HSA05215_PROSTATE_CANCER	46	-0.30302
HSA00564_GLYCEROPHOSPHOLIPID_METABOLISM	22	-0.35368
HSA04670_LEUKOCYTE_TRANSENDOTHELIAL_MIGRATION	55	-0.28758
HSA04210_APOPTOSIS	55	-0.28383
HSA04520_ADHERENS_JUNCTION	39	-0.30279
HSA05212_PANCREATIC_CANCER	41	-0.29992
HSA04060_CYTOKINE_CYTOKINE_RECEPTOR_INTERACTION	66	-0.27417
HSA05213_ENDOMETRIAL_CANCER	29	-0.31457
HSA04916_MELANOGENESIS	34	-0.30404
HSA05218_MELANOMA	22	-0.34385
HSA04910_INSULIN_SIGNALING_PATHWAY	65	-0.27013
HSA04530_TIGHT_JUNCTION	46	-0.2818
HSA00561_GLYCEROLIPID_METABOLISM	17	-0.35212
HSA00410_BETA_ALANINE_METABOLISM	11	-0.4033
HSA04912_GNRH_SIGNALING_PATHWAY	38	-0.28657
HSA05131_PATHOGENIC_ESCHERICHIA_COLI_INFECTION_EPEC	29	-0.30311
HSA00903_LIMONENE_AND_PINENE_DEGRADATION	13	-0.38213
HSA04660_T_CELL_RECEPTOR_SIGNALING_PATHWAY	55	-0.26828
HSA00071_FATTY_ACID_METABOLISM	20	-0.33698
HSA04620_TOLL_LIKE_RECEPTOR_SIGNALING_PATHWAY	49	-0.27323
HSA05210_COLORECTAL_CANCER	42	-0.27984
HSA04720_LONG_TERM_POTENTIATION	37	-0.28123
HSA00230_PURINE_METABOLISM	57	-0.25964
HSA05217_BASAL_CELL_CARCINOMA	12	-0.37633
HSA05110_CHOLERA_INFECTION	25	-0.30767
HSA05130_PATHOGENIC_ESCHERICHIA_COLI_INFECTION_EHEC	29	-0.30311
HSA00480_GLUTATHIONE_METABOLISM	14	-0.35607
HSA03320_PPAR_SIGNALING_PATHWAY	19	-0.32265
HSA03020_RNA_POLYMERASE	13	-0.35677
HSA00632_BENZOATE_DEGRADATION_VIA_COA_LIGATION	11	-0.37251
HSA04640_HEMATOPOIETIC_CELL_LINEAGE	44	-0.25901

HSA04320_DORSO_VENTRAL_AXIS_FORMATION	13	-0.3563
HSA00562_INOSITOL_PHOSPHATE_METABOLISM	22	-0.30247
HSA00310_LYSINE_DEGRADATION	20	-0.30394
HSA05040_HUNTINGTONS_DISEASE	17	-0.3196
HSA04920_ADIPOCYTOKINE_SIGNALING_PATHWAY	38	-0.25857
HSA01032_GLYCAN_STRUCTURES_DEGRADATION	13	-0.34204
HSA00051_FRUCTOSE_AND_MANNOSE_METABOLISM	18	-0.30011
HSA04340_HEDGEHOG_SIGNALING_PATHWAY	11	-0.35173
HSA04110_CELL_CYCLE	57	-0.22934
HSA04740_OLFACTORY_TRANSDUCTION	10	-0.35518
HSA04930_TYPE_II_DIABETES_MELLITUS	14	-0.31962
HSA00020_CITRATE_CYCLE	18	-0.29967
HSA04140_REGULATION_OF_AUTOPHAGY	10	-0.35054
HSA05222_SMALL_CELL_LUNG_CANCER	40	-0.23977
HSA00240_PYRIMIDINE_METABOLISM	34	-0.24156
HSA05216_THYROID_CANCER	17	-0.29267
HSA00280_VALINE_LEUCINE_AND_ISOLEUCINE_DEGRADATION	22	-0.26726
HSA00930_CAPROLACTAM_DEGRADATION	11	-0.32434
HSA00530_AMINOSUGARS_METABOLISM	15	-0.2904
HSA04080_NEUROACTIVE_LIGAND_RECEPTOR_INTERACTION	27	-0.24121
HSA00190_OXIDATIVE_PHOSPHORYLATION	79	-0.19442
HSA04150_MTOR_SIGNALING_PATHWAY	25	-0.23728
HSA04130_SNARE_INTERACTIONS_IN_VESICULAR_TRANSPORT	23	-0.24221
HSA00120_BILE_ACID_BIOSYNTHESIS	15	-0.26861
HSA00650_BUTANOATE_METABOLISM	21	-0.23316
HSA00620_PYRUVATE_METABOLISM	22	-0.22868
HSA02010_ABC_TRANSPORTERS_GENERAL	11	-0.27372
HSA00251_GLUTAMATE_METABOLISM	16	-0.23863
HSA00252_ALANINE_AND_ASPARTATE_METABOLISM	11	-0.26979
HSA04610_COMPLEMENT_AND_COAGULATION_CASCADES	14	-0.24872
HSA04512_ECM_RECEPTOR_INTERACTION	12	-0.24179
HSA00052_GALACTOSE_METABOLISM	12	-0.23348
HSA00220_UREA_CYCLE_AND_METABOLISM_OF_AMINO_GROUPS	10	-0.24945
HSA00500_STARCH_AND_SUCROSE_METABOLISM	24	-0.1862
HSA03050_PROTEASOME	21	-0.18737
HSA00860_PORPHYRIN_AND_CHLOROPHYLL_METABOLISM	13	-0.21493
HSA00260_GLYCINE_SERINE_AND_THREONINE_METABOLISM	13	-0.21031
HSA00960_ALKALOID_BIOSYNTHESIS_II	10	-0.2112
HSA04115_P53_SIGNALING_PATHWAY	32	-0.15071
HSA01031_GLYCAN_STRUCTURES_BIOSYNTHESIS_2	20	-0.1612
HSA04120_UBIQUITIN_MEDIATED_PROTEOLYSIS	25	-0.14955
HSA00512_O_GLYCAN_BIOSYNTHESIS	11	-0.18941
HSA00790_FOLATE_BIOSYNTHESIS	13	-0.17257
HSA00600_SPHINGOLIPID_METABOLISM	12	-0.17579
HSA00563_GLYCOSYLPHOSPHATIDYLINOSITOL_ANCHOR_BIOSYNTHESIS	12	-0.17209

HSA03010_RIBOSOME	54	0.245197
HSA03022_BASAL_TRANSCRIPTION_FACTORS	19	0.348613
HSA04350_TGF_BETA_SIGNALING_PATHWAY	32	0.160695
HSA00100_BIOSYNTHESIS_OF_STEROIDS	10	0.225221
HSA00790_FOLATE_BIOSYNTHESIS	13	0.173971
HSA00563_GLYCOSYLPHOSPHATIDYLINOSITOL_ANCHOR_BIOSYNTHESIS	12	0.165683
HSA04650_NATURAL_KILLER_CELL_MEDIATED_CYTOTOXICITY	72	-0.39302
HSA04612_ANTIGEN_PROCESSING_AND_PRESENTATION	43	-0.42567
HSA05120_EPITHELIAL_CELL_SIGNALING_IN_HELICOBACTER_PYLORI_INFECTION	41	-0.39728
HSA04810_REGULATION_OF_ACTIN_CYTOSKELETON	94	-0.34912
HSA05221_ACUTE_MYELOID_LEUKEMIA	30	-0.43421
HSA05223_NON_SMALL_CELL_LUNG_CANCER	29	-0.43441
HSA04330_NOTCH_SIGNALING_PATHWAY	27	-0.42483
HSA04360_AXON_GUIDANCE	40	-0.38623
HSA04370_VEGF_SIGNALING_PATHWAY	33	-0.39713
HSA04310_WNT_SIGNALING_PATHWAY	67	-0.34166
HSA04630_JAK_STAT_SIGNALING_PATHWAY	58	-0.34839
HSA05215_PROSTATE_CANCER	46	-0.35767
HSA05211_RENAL_CELL_CARCINOMA	39	-0.36851
HSA04010_MAPK_SIGNALING_PATHWAY	103	-0.30863
HSA04510_FOCAL_ADHESION	81	-0.31535
HSA05220_CHRONIC_MYELOID_LEUKEMIA	46	-0.35042
HSA04664_FC_EPSILON_RI_SIGNALING_PATHWAY	39	-0.36095
HSA04520_ADHERENS_JUNCTION	39	-0.35865
HSA05214_GLIOMA	33	-0.3628
HSA01030_GLYCAN_STRUCTURES_BIOSYNTHESIS_1	40	-0.34125
HSA04540_GAP_JUNCTION	31	-0.36344
HSA05131_PATHOGENIC_ESCHERICHIA_COLI_INFECTION_EPEC	29	-0.37043
HSA04670_LEUKOCYTE_TRANSENDOTHELIAL_MIGRATION	55	-0.31867
HSA05219_BLADDER_CANCER	16	-0.44457
HSA05130_PATHOGENIC_ESCHERICHIA_COLI_INFECTION_EHEC	29	-0.37043
HSA04514_CELL_ADHESION_MOLECULES	47	-0.3252
HSA00710_CARBON_FIXATION	10	-0.50383
HSA04012_ERBB_SIGNALING_PATHWAY	43	-0.3291
HSA04660_T_CELL_RECEPTOR_SIGNALING_PATHWAY	55	-0.30292
HSA00010_GLYCOLYSIS_AND_GLUCONEOGENESIS	30	-0.347
HSA04662_B_CELL_RECEPTOR_SIGNALING_PATHWAY	39	-0.32631
HSA05212_PANCREATIC_CANCER	41	-0.32532
HSA04916_MELANOGENESIS	34	-0.33525
HSA04020_CALCIUM_SIGNALING_PATHWAY	50	-0.30444
HSA04912_GNRH_SIGNALING_PATHWAY	38	-0.32595
HSA00970_AMINOACYL_TRNA_BIOSYNTHESIS	20	-0.37327
HSA05213_ENDOMETRIAL_CANCER	29	-0.33842
HSA00030_PENTOSE_PHOSPHATE_PATHWAY	12	-0.44623
HSA04530_TIGHT_JUNCTION	46	-0.30443

HSA05218_MELANOMA	22	-0.35575
HSA05110_CHOLERA_INFECTION	25	-0.33838
HSA04940_TYPE_I_DIABETES_MELLITUS	25	-0.33988
HSA05210_COLORECTAL_CANCER	42	-0.29817
HSA04910_INSULIN_SIGNALING_PATHWAY	65	-0.2697
HSA04140_REGULATION_OF_AUTOPHAGY	10	-0.43468
HSA05216_THYROID_CANCER	17	-0.35198
HSA05217_BASAL_CELL_CARCINOMA	12	-0.40493
HSA04320_DORSO_VENTRAL_AXIS_FORMATION	13	-0.37875
HSA04210_APOPTOSIS	55	-0.25731
HSA00640_PROPANOATE_METABOLISM	18	-0.33655
HSA00564_GLYCEROPHOSPHOLIPID_METABOLISM	22	-0.32207
HSA01510_NEURODEGENERATIVE_DISEASES	22	-0.31209
HSA05222_SMALL_CELL_LUNG_CANCER	40	-0.27015
HSA00510_N_GLYCAN_BIOSYNTHESIS	20	-0.32158
HSA04920_ADIPOCYTOKINE_SIGNALING_PATHWAY	38	-0.26965
HSA04070_PHOSPHATIDYLINOSITOL_SIGNALING_SYSTEM	38	-0.26446
HSA04620_TOLL_LIKE_RECEPTOR_SIGNALING_PATHWAY	49	-0.24752
HSA04720_LONG_TERM_POTENTIATION	37	-0.26562
HSA05040_HUNTINGTONS_DISEASE	17	-0.33012
HSA05030_AMYOTROPHIC_LATERAL_SCLEROSIS	12	-0.36152
HSA00230_PURINE_METABOLISM	57	-0.23775
HSA04060_CYTOKINE_CYTOKINE_RECEPTOR_INTERACTION	66	-0.23256
HSA00340_HISTIDINE_METABOLISM	15	-0.32548
HSA00410_BETA_ALANINE_METABOLISM	11	-0.36367
HSA04730_LONG_TERM_DEPRESSION	24	-0.2886
HSA04340_HEDGEHOG_SIGNALING_PATHWAY	11	-0.35884
HSA00071_FATTY_ACID_METABOLISM	20	-0.29086
HSA03320_PPAR_SIGNALING_PATHWAY	19	-0.29172
HSA00350_TYROSINE_METABOLISM	15	-0.30906
HSA00020_CITRATE_CYCLE	18	-0.27636
HSA00561_GLYCEROLIPID_METABOLISM	17	-0.28434
HSA04930_TYPE_II_DIABETES_MELLITUS	14	-0.30061
HSA00530_AMINOSUGARS_METABOLISM	15	-0.28929
HSA00450_SELENOAMINO_ACID_METABOLISM	11	-0.32234
HSA00220_UREA_CYCLE_AND_METABOLISM_OF_AMINO_GROUPS	10	-0.32489
HSA00190_OXIDATIVE_PHOSPHORYLATION	79	-0.1911
HSA00480_GLUTATHIONE_METABOLISM	14	-0.29369
HSA03020_RNA_POLYMERASE	13	-0.30394
HSA04740_OLFACTORY_TRANSDUCTION	10	-0.3235
HSA04130_SNARE_INTERACTIONS_IN_VESICULAR_TRANSPORT	23	-0.23952
HSA00903_LIMONENE_AND_PINENE_DEGRADATION	13	-0.2806
HSA00562_INOSITOL_PHOSPHATE_METABOLISM	22	-0.23884
HSA00960_ALKALOID_BIOSYNTHESIS_II	10	-0.30906
HSA01032_GLYCAN_STRUCTURES_DEGRADATION	13	-0.28744

HSA04150_MTOR_SIGNALING_PATHWAY	25	-0.23314
HSA04110_CELL_CYCLE	57	-0.18302
HSA00051_FRUCTOSE_AND_MANNOSE_METABOLISM	18	-0.23202
HSA00251_GLUTAMATE_METABOLISM	16	-0.23967
HSA04640_HEMATOPOIETIC_CELL_LINEAGE	44	-0.17833
HSA00280_VALINE_LEUCINE_AND_ISOLEUCINE_DEGRADATION	22	-0.20931
HSA04080_NEUROACTIVE_LIGAND_RECEPTOR_INTERACTION	27	-0.19624
HSA00380_TRYPTOPHAN_METABOLISM	23	-0.19991
HSA00252_ALANINE_AND_ASPARTATE_METABOLISM	11	-0.25021
HSA05010_ALZHEIMERS_DISEASE	11	-0.24589
HSA00930_CAPROLACTAM_DEGRADATION	11	-0.25108
HSA00512_O_GLYCAN_BIOSYNTHESIS	11	-0.24808
HSA03050_PROTEASOME	21	-0.19929
HSA00500_STARCH_AND_SUCROSE_METABOLISM	24	-0.18633
HSA00620_PYRUVATE_METABOLISM	22	-0.18746
HSA00632_BENZOATE_DEGRADATION_VIA_COA_LIGATION	11	-0.23803
HSA00052_GALACTOSE_METABOLISM	12	-0.22632
HSA04512_ECM_RECEPTOR_INTERACTION	12	-0.22753
HSA00650_BUTANOATE_METABOLISM	21	-0.18327
HSA04120_UBIQUITIN_MEDIATED_PROTEOLYSIS	25	-0.17155
HSA02010_ABC_TRANSPORTERS_GENERAL	11	-0.22019
HSA00310_LYSINE_DEGRADATION	20	-0.1806
HSA00120_BILE_ACID_BIOSYNTHESIS	15	-0.19262
HSA00600_SPHINGOLIPID_METABOLISM	12	-0.2017
HSA04115_P53_SIGNALING_PATHWAY	32	-0.14547
HSA00260_GLYCINE_SERINE_AND_THREONINE_METABOLISM	13	-0.1678
HSA04610_COMPLEMENT_AND_COAGULATION_CASCADES	14	-0.16506
HSA00240_PYRIMIDINE_METABOLISM	34	-0.12808
HSA00860_PORPHYRIN_AND_CHLOROPHYLL_METABOLISM	13	-0.16339
HSA01031_GLYCAN_STRUCTURES_BIOSYNTHESIS_2	20	-0.13562

NES	NOM p-va	FDR q-va	FWER p-\	Upregulated in	Cohort
2.36356	0	6.67E-04	0.001	Obese	Adjusted by Erythrocyte Membrane Protein Ba
1.925033	0	0.006681	0.025	Obese	Adjusted by Erythrocyte Membrane Protein Ba
0.944302	0.642857	0.709223	0.985	Obese	Adjusted by Erythrocyte Membrane Protein Ba
0.704485	0.880952	0.882257	1	Obese	Adjusted by Erythrocyte Membrane Protein Ba
-1.89808	0	0.177381	0.158	Lean	Adjusted by Erythrocyte Membrane Protein Ba
-1.8455	0	0.145773	0.245	Lean	Adjusted by Erythrocyte Membrane Protein Ba
-1.82196	0.001111	0.12191	0.302	Lean	Adjusted by Erythrocyte Membrane Protein Ba
-1.81404	0	0.098677	0.321	Lean	Adjusted by Erythrocyte Membrane Protein Ba
-1.80719	0.001003	0.084341	0.34	Lean	Adjusted by Erythrocyte Membrane Protein Ba
-1.78708	0.004086	0.083517	0.386	Lean	Adjusted by Erythrocyte Membrane Protein Ba
-1.75556	0.003128	0.094355	0.467	Lean	Adjusted by Erythrocyte Membrane Protein Ba
-1.73206	0.009685	0.101736	0.54	Lean	Adjusted by Erythrocyte Membrane Protein Ba
-1.71627	0.006079	0.10279	0.587	Lean	Adjusted by Erythrocyte Membrane Protein Ba
-1.6802	0.023121	0.127694	0.707	Lean	Adjusted by Erythrocyte Membrane Protein Ba
-1.66268	0.009269	0.135547	0.76	Lean	Adjusted by Erythrocyte Membrane Protein Ba
-1.65875	0.017544	0.127856	0.771	Lean	Adjusted by Erythrocyte Membrane Protein Ba
-1.65291	0.013514	0.124231	0.793	Lean	Adjusted by Erythrocyte Membrane Protein Ba
-1.63634	0.012183	0.130473	0.83	Lean	Adjusted by Erythrocyte Membrane Protein Ba
-1.60123	0.002	0.161869	0.907	Lean	Adjusted by Erythrocyte Membrane Protein Ba
-1.59038	0.011145	0.164372	0.922	Lean	Adjusted by Erythrocyte Membrane Protein Ba
-1.58957	0.029834	0.155554	0.924	Lean	Adjusted by Erythrocyte Membrane Protein Ba
-1.53749	0.03144	0.21206	0.981	Lean	Adjusted by Erythrocyte Membrane Protein Ba
-1.53601	0.028513	0.20366	0.983	Lean	Adjusted by Erythrocyte Membrane Protein Ba
-1.52956	0.018163	0.201666	0.985	Lean	Adjusted by Erythrocyte Membrane Protein Ba
-1.51169	0.027383	0.21511	0.994	Lean	Adjusted by Erythrocyte Membrane Protein Ba
-1.50356	0.035197	0.21744	0.995	Lean	Adjusted by Erythrocyte Membrane Protein Ba
-1.50168	0.048936	0.211116	0.995	Lean	Adjusted by Erythrocyte Membrane Protein Ba
-1.49664	0.035642	0.209763	0.995	Lean	Adjusted by Erythrocyte Membrane Protein Ba
-1.48996	0.015	0.210963	0.996	Lean	Adjusted by Erythrocyte Membrane Protein Ba
-1.48938	0.038618	0.203756	0.996	Lean	Adjusted by Erythrocyte Membrane Protein Ba

-1.4796	0.052519	0.208671	0.998	Lean	Adjusted by Erythrocyte Membrane Protein Ba
-1.47868	0.053156	0.202321	0.998	Lean	Adjusted by Erythrocyte Membrane Protein Ba
-1.46244	0.045502	0.218554	0.999	Lean	Adjusted by Erythrocyte Membrane Protein Ba
-1.45186	0.039315	0.225261	1	Lean	Adjusted by Erythrocyte Membrane Protein Ba
-1.45038	0.036364	0.22016	1	Lean	Adjusted by Erythrocyte Membrane Protein Ba
-1.44785	0.062433	0.216636	1	Lean	Adjusted by Erythrocyte Membrane Protein Ba
-1.43645	0.086772	0.224877	1	Lean	Adjusted by Erythrocyte Membrane Protein Ba
-1.41706	0.094104	0.246442	1	Lean	Adjusted by Erythrocyte Membrane Protein Ba
-1.41279	0.105856	0.246331	1	Lean	Adjusted by Erythrocyte Membrane Protein Ba
-1.41253	0.053115	0.239858	1	Lean	Adjusted by Erythrocyte Membrane Protein Ba
-1.40849	0.082715	0.238617	1	Lean	Adjusted by Erythrocyte Membrane Protein Ba
-1.40731	0.04509	0.234155	1	Lean	Adjusted by Erythrocyte Membrane Protein Ba
-1.38619	0.106681	0.258106	1	Lean	Adjusted by Erythrocyte Membrane Protein Ba
-1.38379	0.091647	0.255673	1	Lean	Adjusted by Erythrocyte Membrane Protein Ba
-1.38205	0.076536	0.251362	1	Lean	Adjusted by Erythrocyte Membrane Protein Ba
-1.37449	0.086589	0.256625	1	Lean	Adjusted by Erythrocyte Membrane Protein Ba
-1.37014	0.065196	0.257497	1	Lean	Adjusted by Erythrocyte Membrane Protein Ba
-1.35429	0.078708	0.275889	1	Lean	Adjusted by Erythrocyte Membrane Protein Ba
-1.34792	0.096643	0.279052	1	Lean	Adjusted by Erythrocyte Membrane Protein Ba
-1.34119	0.086382	0.28331	1	Lean	Adjusted by Erythrocyte Membrane Protein Ba
-1.32684	0.088442	0.299935	1	Lean	Adjusted by Erythrocyte Membrane Protein Ba
-1.31868	0.134159	0.306491	1	Lean	Adjusted by Erythrocyte Membrane Protein Ba
-1.3099	0.128469	0.315062	1	Lean	Adjusted by Erythrocyte Membrane Protein Ba
-1.30438	0.160127	0.318032	1	Lean	Adjusted by Erythrocyte Membrane Protein Ba
-1.29774	0.107107	0.323208	1	Lean	Adjusted by Erythrocyte Membrane Protein Ba
-1.29275	0.129555	0.325161	1	Lean	Adjusted by Erythrocyte Membrane Protein Ba
-1.28923	0.151251	0.324562	1	Lean	Adjusted by Erythrocyte Membrane Protein Ba
-1.27549	0.17093	0.340542	1	Lean	Adjusted by Erythrocyte Membrane Protein Ba
-1.27527	0.143147	0.334729	1	Lean	Adjusted by Erythrocyte Membrane Protein Ba
-1.27278	0.17562	0.333203	1	Lean	Adjusted by Erythrocyte Membrane Protein Ba
-1.27054	0.176271	0.330725	1	Lean	Adjusted by Erythrocyte Membrane Protein Ba
-1.26944	0.136226	0.326906	1	Lean	Adjusted by Erythrocyte Membrane Protein Ba
-1.26528	0.16973	0.327811	1	Lean	Adjusted by Erythrocyte Membrane Protein Ba
-1.25998	0.15261	0.331258	1	Lean	Adjusted by Erythrocyte Membrane Protein Ba
-1.24946	0.171053	0.342857	1	Lean	Adjusted by Erythrocyte Membrane Protein Ba
-1.24537	0.174717	0.344545	1	Lean	Adjusted by Erythrocyte Membrane Protein Ba
-1.23857	0.152764	0.349744	1	Lean	Adjusted by Erythrocyte Membrane Protein Ba
-1.2337	0.203429	0.351927	1	Lean	Adjusted by Erythrocyte Membrane Protein Ba
-1.22874	0.193111	0.354874	1	Lean	Adjusted by Erythrocyte Membrane Protein Ba
-1.2271	0.197723	0.352283	1	Lean	Adjusted by Erythrocyte Membrane Protein Ba
-1.20581	0.246361	0.38217	1	Lean	Adjusted by Erythrocyte Membrane Protein Ba
-1.20346	0.227224	0.380881	1	Lean	Adjusted by Erythrocyte Membrane Protein Ba
-1.19645	0.236111	0.387014	1	Lean	Adjusted by Erythrocyte Membrane Protein Ba
-1.19602	0.24225	0.382102	1	Lean	Adjusted by Erythrocyte Membrane Protein Ba
-1.17206	0.251509	0.417875	1	Lean	Adjusted by Erythrocyte Membrane Protein Ba

-1.17157	0.278613	0.413044	1	Lean	Adjusted by Erythrocyte Membrane Protein Ba
-1.16869	0.260963	0.411899	1	Lean	Adjusted by Erythrocyte Membrane Protein Ba
-1.16153	0.272727	0.418271	1	Lean	Adjusted by Erythrocyte Membrane Protein Ba
-1.15371	0.282468	0.426315	1	Lean	Adjusted by Erythrocyte Membrane Protein Ba
-1.13635	0.277268	0.451703	1	Lean	Adjusted by Erythrocyte Membrane Protein Ba
-1.12488	0.316327	0.466675	1	Lean	Adjusted by Erythrocyte Membrane Protein Ba
-1.10645	0.343137	0.494437	1	Lean	Adjusted by Erythrocyte Membrane Protein Ba
-1.10488	0.351536	0.491433	1	Lean	Adjusted by Erythrocyte Membrane Protein Ba
-1.09761	0.353119	0.498114	1	Lean	Adjusted by Erythrocyte Membrane Protein Ba
-1.09693	0.339557	0.49302	1	Lean	Adjusted by Erythrocyte Membrane Protein Ba
-1.09599	0.355758	0.48857	1	Lean	Adjusted by Erythrocyte Membrane Protein Ba
-1.09545	0.340195	0.483773	1	Lean	Adjusted by Erythrocyte Membrane Protein Ba
-1.09132	0.366627	0.485238	1	Lean	Adjusted by Erythrocyte Membrane Protein Ba
-1.05565	0.390418	0.54385	1	Lean	Adjusted by Erythrocyte Membrane Protein Ba
-1.05463	0.405128	0.539269	1	Lean	Adjusted by Erythrocyte Membrane Protein Ba
-1.04699	0.418831	0.54687	1	Lean	Adjusted by Erythrocyte Membrane Protein Ba
-1.03865	0.429654	0.555479	1	Lean	Adjusted by Erythrocyte Membrane Protein Ba
-1.02856	0.441725	0.567223	1	Lean	Adjusted by Erythrocyte Membrane Protein Ba
-1.01464	0.449048	0.586392	1	Lean	Adjusted by Erythrocyte Membrane Protein Ba
-0.98348	0.502092	0.634768	1	Lean	Adjusted by Erythrocyte Membrane Protein Ba
-0.98153	0.514056	0.631574	1	Lean	Adjusted by Erythrocyte Membrane Protein Ba
-0.95167	0.5327	0.678175	1	Lean	Adjusted by Erythrocyte Membrane Protein Ba
-0.944	0.536741	0.685299	1	Lean	Adjusted by Erythrocyte Membrane Protein Ba
-0.94244	0.546682	0.680989	1	Lean	Adjusted by Erythrocyte Membrane Protein Ba
-0.89848	0.628571	0.748151	1	Lean	Adjusted by Erythrocyte Membrane Protein Ba
-0.88228	0.644585	0.768013	1	Lean	Adjusted by Erythrocyte Membrane Protein Ba
-0.86498	0.641355	0.789651	1	Lean	Adjusted by Erythrocyte Membrane Protein Ba
-0.85185	0.645949	0.802044	1	Lean	Adjusted by Erythrocyte Membrane Protein Ba
-0.84997	0.660998	0.796654	1	Lean	Adjusted by Erythrocyte Membrane Protein Ba
-0.84089	0.675339	0.802588	1	Lean	Adjusted by Erythrocyte Membrane Protein Ba
-0.79144	0.724256	0.867767	1	Lean	Adjusted by Erythrocyte Membrane Protein Ba
-0.7779	0.741163	0.877512	1	Lean	Adjusted by Erythrocyte Membrane Protein Ba
-0.7622	0.781775	0.890426	1	Lean	Adjusted by Erythrocyte Membrane Protein Ba
-0.73679	0.800848	0.913903	1	Lean	Adjusted by Erythrocyte Membrane Protein Ba
-0.72204	0.826638	0.921953	1	Lean	Adjusted by Erythrocyte Membrane Protein Ba
-0.71644	0.826087	0.919533	1	Lean	Adjusted by Erythrocyte Membrane Protein Ba
-0.71	0.84009	0.917836	1	Lean	Adjusted by Erythrocyte Membrane Protein Ba
-0.65546	0.881414	0.961398	1	Lean	Adjusted by Erythrocyte Membrane Protein Ba
-0.64555	0.907217	0.960887	1	Lean	Adjusted by Erythrocyte Membrane Protein Ba
-0.60969	0.933405	0.978132	1	Lean	Adjusted by Erythrocyte Membrane Protein Ba
-0.60163	0.930777	0.97447	1	Lean	Adjusted by Erythrocyte Membrane Protein Ba
-0.59819	0.94269	0.96794	1	Lean	Adjusted by Erythrocyte Membrane Protein Ba
-0.56838	0.948689	0.975855	1	Lean	Adjusted by Erythrocyte Membrane Protein Ba
-0.56517	0.948081	0.968981	1	Lean	Adjusted by Erythrocyte Membrane Protein Ba
-0.56495	0.952656	0.96076	1	Lean	Adjusted by Erythrocyte Membrane Protein Ba

1.889966	0	0.040328	0.06 Obese	Adjusted by HemoglobinD
1.857999	0	0.024671	0.071 Obese	Adjusted by HemoglobinD
1.082684	0.288889	0.663616	0.96 Obese	Adjusted by HemoglobinD
0.892636	0.601156	0.901586	0.996 Obese	Adjusted by HemoglobinD
0.754762	0.788321	0.97994	0.999 Obese	Adjusted by HemoglobinD
0.688047	0.872483	0.896864	0.999 Obese	Adjusted by HemoglobinD
-2.07309	0	0.043401	0.041 Lean	Adjusted by HemoglobinD
-2.05378	0	0.024727	0.047 Lean	Adjusted by HemoglobinD
-1.94824	0	0.049353	0.132 Lean	Adjusted by HemoglobinD
-1.93029	0	0.043965	0.152 Lean	Adjusted by HemoglobinD
-1.92749	0.002101	0.035572	0.154 Lean	Adjusted by HemoglobinD
-1.91076	0.001062	0.033591	0.173 Lean	Adjusted by HemoglobinD
-1.85132	0.004197	0.052042	0.299 Lean	Adjusted by HemoglobinD
-1.81919	0.00103	0.056514	0.365 Lean	Adjusted by HemoglobinD
-1.80491	0.005192	0.05834	0.416 Lean	Adjusted by HemoglobinD
-1.79805	0.001005	0.055266	0.431 Lean	Adjusted by HemoglobinD
-1.79304	0	0.052281	0.441 Lean	Adjusted by HemoglobinD
-1.76394	0.002035	0.061918	0.539 Lean	Adjusted by HemoglobinD
-1.73903	0.004128	0.069281	0.614 Lean	Adjusted by HemoglobinD
-1.72685	0.001	0.071782	0.652 Lean	Adjusted by HemoglobinD
-1.71561	0.004004	0.072816	0.682 Lean	Adjusted by HemoglobinD
-1.70904	0.004082	0.072246	0.702 Lean	Adjusted by HemoglobinD
-1.69902	0.007194	0.074631	0.724 Lean	Adjusted by HemoglobinD
-1.68158	0.009298	0.08143	0.772 Lean	Adjusted by HemoglobinD
-1.66378	0.017526	0.089686	0.826 Lean	Adjusted by HemoglobinD
-1.65911	0.010235	0.08825	0.841 Lean	Adjusted by HemoglobinD
-1.64328	0.015707	0.095653	0.876 Lean	Adjusted by HemoglobinD
-1.63828	0.012698	0.094312	0.884 Lean	Adjusted by HemoglobinD
-1.63415	0.011122	0.092663	0.891 Lean	Adjusted by HemoglobinD
-1.63394	0.035477	0.088928	0.891 Lean	Adjusted by HemoglobinD
-1.62715	0.020474	0.089981	0.905 Lean	Adjusted by HemoglobinD
-1.62113	0.015275	0.0902	0.915 Lean	Adjusted by HemoglobinD
-1.60067	0.02843	0.100988	0.946 Lean	Adjusted by HemoglobinD
-1.59542	0.018311	0.100982	0.954 Lean	Adjusted by HemoglobinD
-1.56027	0.027163	0.123115	0.98 Lean	Adjusted by HemoglobinD
-1.55266	0.035565	0.125737	0.985 Lean	Adjusted by HemoglobinD
-1.53832	0.028513	0.134328	0.993 Lean	Adjusted by HemoglobinD
-1.53009	0.033639	0.137938	0.995 Lean	Adjusted by HemoglobinD
-1.52601	0.033473	0.138013	0.996 Lean	Adjusted by HemoglobinD
-1.52302	0.028455	0.136323	0.996 Lean	Adjusted by HemoglobinD
-1.52026	0.038263	0.134666	0.996 Lean	Adjusted by HemoglobinD
-1.51657	0.060976	0.134046	0.997 Lean	Adjusted by HemoglobinD
-1.49161	0.052138	0.153288	0.999 Lean	Adjusted by HemoglobinD
-1.47932	0.071429	0.161919	0.999 Lean	Adjusted by HemoglobinD
-1.46625	0.030581	0.17239	0.999 Lean	Adjusted by HemoglobinD

-1.44773	0.075269	0.185997	1	Lean	Adjusted by HemoglobinD
-1.43789	0.075132	0.192329	1	Lean	Adjusted by HemoglobinD
-1.43715	0.068182	0.188556	1	Lean	Adjusted by HemoglobinD
-1.42339	0.07582	0.199232	1	Lean	Adjusted by HemoglobinD
-1.41002	0.055444	0.211499	1	Lean	Adjusted by HemoglobinD
-1.37977	0.107748	0.245677	1	Lean	Adjusted by HemoglobinD
-1.34659	0.121924	0.2848	1	Lean	Adjusted by HemoglobinD
-1.33605	0.151899	0.295355	1	Lean	Adjusted by HemoglobinD
-1.32463	0.147541	0.306565	1	Lean	Adjusted by HemoglobinD
-1.32073	0.119839	0.305907	1	Lean	Adjusted by HemoglobinD
-1.31679	0.147415	0.305862	1	Lean	Adjusted by HemoglobinD
-1.31583	0.157895	0.301277	1	Lean	Adjusted by HemoglobinD
-1.28814	0.181128	0.341346	1	Lean	Adjusted by HemoglobinD
-1.27904	0.159346	0.350258	1	Lean	Adjusted by HemoglobinD
-1.27142	0.174058	0.356482	1	Lean	Adjusted by HemoglobinD
-1.26194	0.18413	0.366474	1	Lean	Adjusted by HemoglobinD
-1.24923	0.184156	0.381533	1	Lean	Adjusted by HemoglobinD
-1.24108	0.18246	0.38916	1	Lean	Adjusted by HemoglobinD
-1.23854	0.19938	0.387041	1	Lean	Adjusted by HemoglobinD
-1.23615	0.213808	0.38489	1	Lean	Adjusted by HemoglobinD
-1.22803	0.224852	0.39213	1	Lean	Adjusted by HemoglobinD
-1.21803	0.197581	0.402747	1	Lean	Adjusted by HemoglobinD
-1.21555	0.184925	0.400173	1	Lean	Adjusted by HemoglobinD
-1.19757	0.247166	0.42438	1	Lean	Adjusted by HemoglobinD
-1.19366	0.24228	0.424714	1	Lean	Adjusted by HemoglobinD
-1.18322	0.268319	0.436786	1	Lean	Adjusted by HemoglobinD
-1.16489	0.277443	0.462949	1	Lean	Adjusted by HemoglobinD
-1.15502	0.281046	0.47413	1	Lean	Adjusted by HemoglobinD
-1.15235	0.289855	0.472064	1	Lean	Adjusted by HemoglobinD
-1.11282	0.320269	0.538205	1	Lean	Adjusted by HemoglobinD
-1.07559	0.373184	0.605007	1	Lean	Adjusted by HemoglobinD
-1.07052	0.40226	0.606777	1	Lean	Adjusted by HemoglobinD
-1.05917	0.384793	0.621547	1	Lean	Adjusted by HemoglobinD
-1.04532	0.431054	0.640947	1	Lean	Adjusted by HemoglobinD
-1.04509	0.405882	0.632595	1	Lean	Adjusted by HemoglobinD
-1.03829	0.400494	0.637232	1	Lean	Adjusted by HemoglobinD
-1.03692	0.420261	0.631511	1	Lean	Adjusted by HemoglobinD
-1.03677	0.411696	0.623709	1	Lean	Adjusted by HemoglobinD
-1.03661	0.407801	0.616028	1	Lean	Adjusted by HemoglobinD
-1.03067	0.420147	0.619599	1	Lean	Adjusted by HemoglobinD
-0.99017	0.480645	0.689722	1	Lean	Adjusted by HemoglobinD
-0.9881	0.476636	0.6848	1	Lean	Adjusted by HemoglobinD
-0.98736	0.469388	0.67779	1	Lean	Adjusted by HemoglobinD
-0.98092	0.470874	0.681317	1	Lean	Adjusted by HemoglobinD
-0.98029	0.487239	0.674567	1	Lean	Adjusted by HemoglobinD

-0.97702	0.507415	0.672426	1	Lean	Adjusted by HemoglobinD
-0.94328	0.56754	0.727706	1	Lean	Adjusted by HemoglobinD
-0.89124	0.628635	0.815527	1	Lean	Adjusted by HemoglobinD
-0.87508	0.634637	0.835995	1	Lean	Adjusted by HemoglobinD
-0.86208	0.682329	0.849937	1	Lean	Adjusted by HemoglobinD
-0.85145	0.655738	0.858405	1	Lean	Adjusted by HemoglobinD
-0.83007	0.701604	0.884413	1	Lean	Adjusted by HemoglobinD
-0.82882	0.702151	0.876737	1	Lean	Adjusted by HemoglobinD
-0.82647	0.711058	0.870929	1	Lean	Adjusted by HemoglobinD
-0.82307	0.704276	0.866702	1	Lean	Adjusted by HemoglobinD
-0.82025	0.692488	0.862073	1	Lean	Adjusted by HemoglobinD
-0.81856	0.70267	0.855487	1	Lean	Adjusted by HemoglobinD
-0.79301	0.755459	0.884746	1	Lean	Adjusted by HemoglobinD
-0.78912	0.749732	0.880933	1	Lean	Adjusted by HemoglobinD
-0.7747	0.768649	0.892898	1	Lean	Adjusted by HemoglobinD
-0.77435	0.767497	0.884564	1	Lean	Adjusted by HemoglobinD
-0.77213	0.753278	0.878892	1	Lean	Adjusted by HemoglobinD
-0.75678	0.772358	0.890822	1	Lean	Adjusted by HemoglobinD
-0.74212	0.800871	0.901332	1	Lean	Adjusted by HemoglobinD
-0.72623	0.825027	0.911533	1	Lean	Adjusted by HemoglobinD
-0.72316	0.824437	0.906531	1	Lean	Adjusted by HemoglobinD
-0.70566	0.843681	0.917738	1	Lean	Adjusted by HemoglobinD
-0.68968	0.851016	0.924971	1	Lean	Adjusted by HemoglobinD
-0.6818	0.845972	0.924353	1	Lean	Adjusted by HemoglobinD
-0.64756	0.906832	0.946314	1	Lean	Adjusted by HemoglobinD
-0.59768	0.933174	0.973573	1	Lean	Adjusted by HemoglobinD
-0.58867	0.928654	0.970241	1	Lean	Adjusted by HemoglobinD
-0.5757	0.947585	0.969537	1	Lean	Adjusted by HemoglobinD
-0.57012	0.958097	0.96358	1	Lean	Adjusted by HemoglobinD
-0.53101	0.978166	0.972842	1	Lean	Adjusted by HemoglobinD

and 2 (EMPB2)
and 2 (EMPB2)
and 2 (EMPB2)
and 2 (EMPB2)
and 2 (EMPB2)
and 2 (EMPB2)
and 2 (EMPB2)
and 2 (EMPB2)
and 2 (EMPB2)
and 2 (EMPB2)
and 2 (EMPB2)
and 2 (EMPB2)
and 2 (EMPB2)
and 2 (EMPB2)
and 2 (EMPB2)
and 2 (EMPB2)
and 2 (EMPB2)
and 2 (EMPB2)
and 2 (EMPB2)
and 2 (EMPB2)
and 2 (EMPB2)
and 2 (EMPB2)
and 2 (EMPB2)
and 2 (EMPB2)
and 2 (EMPB2)
and 2 (EMPB2)
and 2 (EMPB2)
and 2 (EMPB2)
and 2 (EMPB2)
and 2 (EMPB2)

and 2 (EMPB2)
and 2 (EMPB2)
and 2 (EMPB2)
and 2 (EMPB2)
and 2 (EMPB2)
and 2 (EMPB2)
and 2 (EMPB2)
and 2 (EMPB2)
and 2 (EMPB2)
and 2 (EMPB2)
and 2 (EMPB2)
and 2 (EMPB2)
and 2 (EMPB2)
and 2 (EMPB2)
and 2 (EMPB2)
and 2 (EMPB2)
and 2 (EMPB2)
and 2 (EMPB2)
and 2 (EMPB2)
and 2 (EMPB2)
and 2 (EMPB2)
and 2 (EMPB2)
and 2 (EMPB2)
and 2 (EMPB2)
and 2 (EMPB2)
and 2 (EMPB2)
and 2 (EMPB2)
and 2 (EMPB2)
and 2 (EMPB2)
and 2 (EMPB2)
and 2 (EMPB2)
and 2 (EMPB2)
and 2 (EMPB2)
and 2 (EMPB2)
and 2 (EMPB2)
and 2 (EMPB2)
and 2 (EMPB2)
and 2 (EMPB2)
and 2 (EMPB2)
and 2 (EMPB2)
and 2 (EMPB2)
and 2 (EMPB2)
and 2 (EMPB2)
and 2 (EMPB2)
and 2 (EMPB2)

and 2 (EMPB2)
and 2 (EMPB2)
and 2 (EMPB2)
and 2 (EMPB2)
and 2 (EMPB2)
and 2 (EMPB2)
and 2 (EMPB2)
and 2 (EMPB2)
and 2 (EMPB2)
and 2 (EMPB2)
and 2 (EMPB2)
and 2 (EMPB2)
and 2 (EMPB2)
and 2 (EMPB2)
and 2 (EMPB2)
and 2 (EMPB2)
and 2 (EMPB2)
and 2 (EMPB2)
and 2 (EMPB2)
and 2 (EMPB2)
and 2 (EMPB2)
and 2 (EMPB2)
and 2 (EMPB2)
and 2 (EMPB2)
and 2 (EMPB2)
and 2 (EMPB2)
and 2 (EMPB2)
and 2 (EMPB2)
and 2 (EMPB2)
and 2 (EMPB2)
and 2 (EMPB2)
and 2 (EMPB2)
and 2 (EMPB2)
and 2 (EMPB2)
and 2 (EMPB2)
and 2 (EMPB2)
and 2 (EMPB2)
and 2 (EMPB2)
and 2 (EMPB2)
and 2 (EMPB2)
and 2 (EMPB2)
and 2 (EMPB2)
and 2 (EMPB2)
and 2 (EMPB2)
and 2 (EMPB2)
