# Supplementary material for: Gene expression profiling in whole blood identifies distinct biological pathways associated with obesity
Source: BMC Med Genomics. 2010 Dec 1;3:56. doi: 10.1186/1755-8794-3-56 (PMC3014865; doi:10.1186/1755-8794-3-56)
Supplement: Additional file 14 — Description of classifiers and classifier performance evaluators used in the study. Brief descriptions of the Naïve Bayes, Logistic Regression, Random Forests and ZeroR classifiers along with the feature selection algorithms used (Information Gain and Cfs Subset evaluator) are given. Mathematical formulas for true and false positive and negative rates (classifier evaluation metrics) are also provided. [file 1755-8794-3-56-S14.DOC]

**Additional File 12**

Description of classifiers and feature-selection algorithms

| **Classifier** | **Description** |
| --- | --- |
| Naïve Bayes | Naive-Bayes is based on the Bayesian theorem. This classification technique analyses the relationship between each attribute and the class for each instance to derive a conditional probability for the relationships between the attribute values and the class. Naïve Bayesian classifiers estimate the probabilities of a feature having a certain feature value. |
| Logistic Regression | The logistic regression model uses the predictor variables (categorical or continuous) to predict the probability of specific outcomes. To ensure that the estimated probabilities are between 0 and 1 and since the typical relationship between the probability of the outcome and a predictor variable is nonlinear (S-shaped) rather than linear, logistic regression transforms the probabilities (p) with the logit transformation (logit(p) = log(p/(1-p)) (where log is the natural logarithm). |
| Random Forest | A Random Forest classifier consists of many decision trees and outputs the class that is the mode of the classes output by individual trees. |
| ZeroR | ZeroR classifier simply predicts the majority class in the training data. Although it makes little sense to use this scheme for prediction, it can be useful for determining a baseline  performance as a benchmark for other learning schemes. |
|  |  |
| **Feature Evaluator** | **Description** |
| Cfs subset evaluator with BestFirst search method | Evaluates the worth of a subset of features by considering the individual predictive ability of each feature along with the degree of redundancy between them; subsets of features that are highly correlated with the class while having low inter-correlation are preferred. The BestFirst search method searches the space of feature subsets by greedy hill-climbing augmented with a backtracking facility. |
| Information Gain with Ranker | Evaluates the worth of a feature by measuring the information gain with respect to the class. The Ranker method ranks features by their individual evaluations |

CLASSIFIER PERFORMANCE MEASURES

|  |  | **True phenotype** | |
| --- | --- | --- | --- |
|  |  | **Obese** | **Lean** |
| **Predicted phenotype** | **Obese** | A | B |
|  | **Lean** | C | D |

**True positive rate (sensitivity) = A/(A+C)**

**True negative rate (specificity) = D/(B+D)**

**False positive rate (1-specificity) = B/(B+D)**

**False negative rate (1-sensitivity) = C/(A+C)**
